# Soulangianolide A targets FAM120A to disrupt SREBP1-driven lipid metabolic reprogramming and suppress clear cell renal cell carcinoma

**DOI:** 10.1186/s13046-026-03788-2

**Published:** 2026-07-17

**Authors:** Wei Luo, Zheng Song, Zishu Liu, Guangshuai Zhang, Si Yan, Rui Peng, Zhaofang Bai, Shuanglin Qin, Xiaohe Xiao

**Affiliations:** 1 School of Pharmacy, Research Center for Precision Medication of Chinese Medicine, Hunan University of Chinese Medicine, Changsha, China; 2 Institute of Urology, Peking University First Hospital, Beijing, China; 3 Department of Hepatology, The Fifth Medical Center of PLA General Hospital, Beijing, China

**Keywords:** Clear cell renal cell carcinoma, FAM120A, SREBP1, Soulangianolide A, Lipid metabolic reprogramming

## Abstract

**Background:**

Clear cell renal cell carcinoma (ccRCC) is characterized by extensive lipid metabolic reprogramming that drives tumor progression and therapeutic resistance. Although sterol regulatory element-binding protein 1 (SREBP1) is a central regulator of lipogenesis in ccRCC, the upstream mechanisms governing its activation remain poorly defined. Here, we investigated the role of FAM120A in SREBP1-dependent lipid metabolism and evaluated the therapeutic potential of the natural product Soulangianolide A (SA).

**Methods:**

Stable knockdown of SREBP1 or FAM120A was established in ccRCC cells to assess lipid metabolism and malignant phenotypes. The antitumor activity and molecular mechanism of SA were investigated using integrated lipidomic, transcriptomic, proteomic, biochemical, and xenograft analyses, together with molecular docking, pull-down assays, surface plasmon resonance, and cellular thermal shift assays.

**Results:**

Genetic depletion of SREBP1 suppressed lipogenic gene expression, intracellular lipid accumulation, and malignant phenotypes in ccRCC cells. FAM120A promoted SREBP1 nuclear accumulation and transcriptional activity, whereas SA inhibited SREBP1 signaling, reduced lipid storage, and induced apoptosis. Multi-omics analyses revealed extensive metabolic remodeling characterized by depletion of triglycerides and membrane lipids, accompanied by accumulation of ceramides and acylcarnitines, indicative of a lipotoxic state. Mechanistically, SA directly bound FAM120A at Cys531, disrupted the FAM120A-SREBP1 complex, impaired SREBP1 nuclear activity, and consequently attenuated lipid-dependent PPARγ signaling. In xenograft models, SA significantly suppressed tumor growth, reduced intratumoral lipid accumulation, inhibited SREBP1-driven lipogenic signaling, and potentiated the antitumor efficacy of MK-6482.

**Conclusions:**

Our findings identify the FAM120A-SREBP1-PPARγ axis as a critical driver of lipid metabolic reprogramming in ccRCC, establish FAM120A as a direct molecular target of SA, and demonstrate that pharmacological disruption of this pathway effectively suppresses tumor growth. These findings provide a mechanistic rationale for targeting lipid metabolic reprogramming in ccRCC and support SA as a promising metabolism-targeted therapeutic strategy.

**Supplementary Information:**

The online version contains supplementary material available at https://doi.org/10.1186/s13046-026-03788-2.

## Introduction

Clear cell renal cell carcinoma (ccRCC), the predominant histological subtype of renal cell carcinoma, is characterized by aggressive progression and poor clinical outcomes in advanced-stage disease [1–3]. A defining feature of ccRCC is the excessive accumulation of neutral lipids, which gives rise to its characteristic “clear cell” morphology and reflects profound lipid metabolic reprogramming [4, 5]. This metabolic phenotype not only supports rapid tumor growth and survival but also promotes therapeutic resistance and immune evasion [6–8]. Although targeted therapies and immune checkpoint inhibitors have substantially improved the treatment landscape of advanced ccRCC, durable clinical responses remain limited by acquired resistance [9, 10], underscoring the need for therapeutic strategies that target metabolic vulnerabilities.

Sterol regulatory element-binding protein 1 (SREBP1) is a master transcription factor that orchestrates de novo lipogenesis by activating genes involved in fatty acid and cholesterol biosynthesis [11]. Aberrant SREBP1 activation has been implicated in the progression of multiple malignancies, including hepatocellular, gastric, pancreatic, and bladder cancers [11–13]. Upon proteolytic processing and nuclear translocation, mature SREBP1 initiates lipogenic transcriptional programs that facilitate tumor growth and metabolic adaptation [14–16]. Despite its central role in lipid metabolism, the upstream mechanisms that sustain SREBP1 activation in ccRCC remain largely undefined.

Family with sequence similarity 120 A (FAM120A) is a multifunctional scaffold protein involved in RNA processing, cellular stress responses, and metabolic regulation [17]. Recent studies have implicated FAM120A in adipogenesis and lipid homeostasis through modulation of adipogenic gene expression [18]. However, whether FAM120A regulates SREBP1-dependent lipogenic transcription and thereby contributes to lipid metabolic reprogramming in ccRCC has not been explored. Given its emerging role in lipid homeostasis, FAM120A may represent an upstream regulator of lipogenic transcriptional networks. Elucidating the upstream regulators of SREBP1 may therefore uncover new mechanisms driving metabolic remodeling and identify actionable therapeutic targets for ccRCC.

Natural products constitute an important source of anticancer agents and frequently provide unique chemical scaffolds for targeting oncogenic pathways [19, 20]. Soulangianolide A (SA), a sesquiterpene lactone isolated from *Saussurea costus* (Falc.) Lipsch., has been reported to possess anti-inflammatory and antitumor activities [21]. However, its direct molecular targets and metabolic mechanisms of action in ccRCC remain unknown.

In this study, we identify FAM120A as a critical upstream regulator that sustains SREBP1-dependent lipogenic signaling in ccRCC. We further demonstrate that SA covalently binds FAM120A at Cys531, disrupts the FAM120A-SREBP1 complex, and suppresses SREBP1-mediated lipid metabolic reprogramming, thereby inducing lipotoxic stress, apoptosis, and tumor growth inhibition. Moreover, we establish the FAM120A-SREBP1-PPARγ axis as a key metabolic pathway underlying ccRCC progression and provide mechanistic evidence supporting FAM120A-targeted metabolic intervention as a promising therapeutic strategy, either alone or in combination with HIF-2α inhibition.

## Materials and methods

### Cell culture

Human clear cell renal cell carcinoma cell lines 786-O and Caki-1 were cultured in Dulbecco’s Modified Eagle Medium supplemented with 10% fetal bovine serum and 1% penicillin–streptomycin at 37 °C in a humidified atmosphere containing 5% CO₂. SA was synthesized in our laboratory and structurally verified by nuclear magnetic resonance (NMR) analysis. SA was dissolved in dimethyl sulfoxide as a stock solution. MK-6482 (HY-125840), Oleic acid (HY-N1446), and Linoleic acid (HY-N0729) were purchased from MedChemExpress and prepared according to the manufacturer’s instructions.

### Plasmids and Lentiviral transduction

Short hairpin RNAs (shRNAs) targeting human SREBP1 (shSREBP1#1 and shSREBP1#2) and FAM120A (shFAM120A#1 and shFAM120A#2), the corresponding overexpression plasmids, and a non-targeting control shRNA (shNC) were purchased from Beijing Boran Yixin Biotechnology Co., Ltd. Lentiviral transduction was performed according to the manufacturer’s instructions, and stable cell lines were established by puromycin selection.

### Cell viability assay

Cell viability was determined using the Cell Counting Kit-8 (CCK-8; Dojindo) according to the manufacturer’s instructions. Absorbance was measured at 450 nm using a microplate reader.

### EdU incorporation assay

Cells were seeded into 12-well plates and treated with the indicated concentrations of SA for 12 h. Cell proliferation was evaluated using an EdU Cell Proliferation Detection Kit according to the manufacturer’s instructions. Fluorescence images were captured using a fluorescence microscope.

### Cell cycle and Annexin V-FITC/PI apoptosis assay

For cell cycle analysis, cells were treated with SA for 12 h, collected, fixed, stained using a Cell Cycle Analysis Kit, and analyzed by flow cytometry. For apoptosis analysis, cells were treated with SA for 12 h and stained using an Annexin V-FITC/PI Apoptosis Detection Kit according to the manufacturer’s protocol before flow cytometric analysis.

### Colony formation assay

786-O and Caki-1 cells (1 × 10³ cells/well) were seeded into six-well plates and cultured in the presence of SA. The medium was replaced every 3 days. After 14 days, colonies were fixed with methanol for 30 min, stained with 0.05% crystal violet for 20 min, photographed, and quantified using ImageJ software.

### Cell thermal shift assay (CETSA)

Cell lysates from 786-O and Caki-1 cells were incubated with SA (10 µM) or DMSO for 2 h at room temperature and heated at the indicated temperatures for 3 min. After centrifugation, supernatants were collected, mixed with 5× loading buffer, boiled for 5 min, and analyzed by immunoblotting.

### Molecular docking

The molecular structure of SA was obtained from the PubChem database. FAM120A was prepared using the Protonate 3D and Energy Minimize modules in Molecular Operating Environment (MOE) software. Molecular docking was performed using the Induced Fit protocol in the DOCK module with the original ligand-binding pocket defined as the docking site.

### RT-qPCR

Total RNA was extracted using TRIzol reagent and reverse-transcribed into cDNA using SuperMix for qPCR. Quantitative PCR was performed using SYBR Green Master Mix, and gene expression levels were normalized to GAPDH and calculated using the 2 − ΔΔCt method. Primer sequences are listed in Supplementary Table S1. CUT&Tag-qPCR was performed as previously described [13].

### Western blotting and immunofluorescence

Total protein was extracted using lysis buffer and quantified by the BCA method. Equal amounts of protein were separated by SDS-PAGE, transferred onto PVDF membranes, and incubated with primary antibodies overnight at 4 °C followed by HRP-conjugated secondary antibodies. Protein bands were visualized using a GelView6000Pro II imaging system. Antibody information is provided in Supplementary Table S2. For immunofluorescence analysis, cells were fixed, permeabilized, and incubated with primary antibodies against SREBP1 or PPARγ followed by fluorescent secondary antibodies. Nuclei were counterstained with DAPI, and images were acquired using a fluorescence microscope.

### Transwell invasion assay

Transwell inserts were precoated with Matrigel (1:8 dilution). After serum starvation for 24 h, cells were seeded into the upper chamber in the presence or absence of SA, while medium containing 10% FBS was added to the lower chamber. After 24 h, invaded cells were fixed, stained with crystal violet, and imaged.

### Measurement of triglycerides and total cholesterol

Cells were treated with SA for 12 h, lysed in 2% Triton X-100, and analyzed using triglyceride and total cholesterol assay kits (Nanjing Jiancheng). Absorbance was measured at 546 nm.

### Oil red O staining

Following SA treatment, cells were fixed with 4% paraformaldehyde and stained with Oil Red O working solution for 1 h at room temperature. Lipid droplets were visualized by light microscopy.

### Surface Plasmon Resonance (SPR) analysis

SPR experiments were performed using a Biacore T200 instrument (GE Healthcare) equipped with a CM5 sensor chip at 37 °C. Recombinant human FAM120A protein (50 µg/mL) was immobilized on the sensor chip, and serial concentrations of SA were injected in running buffer. Binding kinetics and equilibrium dissociation constants (KD) were calculated using Biacore T200 Evaluation Software.

### Untargeted lipidomics analysis

Untargeted lipidomic profiling was performed by Guangdong Meige Gene Technology Co., Ltd. (Guangdong, China). Lipid metabolites were extracted using a chloroform/methanol/water extraction protocol and analyzed by ultra-high-performance liquid chromatography coupled with tandem mass spectrometry (UHPLC-MS/MS).

### Proteomic and transcriptomic analyses

Proteomic analysis was performed by Shanghai Zhongke Xinlife Biotechnology Co., Ltd. (Shanghai, China). Differentially expressed proteins were identified using a significance threshold of *P* < 0.05 and subjected to GO and KEGG pathway enrichment analyses, hierarchical clustering, and volcano plot analysis.

Transcriptomic sequencing was performed by Shanghai Bohao Biotechnology Co., Ltd. (Shanghai, China). Differentially expressed genes were defined as those with |log₂FoldChange| > 1 and *P* < 0.05.

### Immunoprecipitation and pull-down assays

Immunoprecipitation and pull-down assays were performed as previously described [22]. Briefly, cell lysates containing protease inhibitors (GenStar, E127) were incubated overnight at 4 °C with SA-conjugated Sepharose 4B beads. After extensive washing, bound proteins were analyzed by western blotting. For co-immunoprecipitation, cell lysates were incubated with the indicated antibodies overnight at 4 °C followed by protein A/G agarose beads.

### LC-MS/MS analysis of SA binding sites

Recombinant human FAM120A protein (amino acids 411–560) was incubated with Soulangianolide A (SA) under the indicated conditions, separated by SDS–PAGE, and subjected to in-gel digestion using Trypsin/Lys-C. The resulting peptides were analyzed by nanoLC–MS/MS on an UltiMate 3000 RSLCnano system coupled to an Orbitrap Fusion Lumos mass spectrometer (Thermo Fisher Scientific). MS/MS data were processed using Proteome Discoverer (Thermo Fisher Scientific) with a custom variable modification corresponding to the SA adduct. The modified peptide and modification site were assigned based on database-supported MS/MS fragment ion matching and manual inspection of representative fragment ions. The identified modified cysteine residue (Cys121 in the recombinant fragment) corresponds to Cys531 in full-length human FAM120A.

### Nuclear and cytoplasmic fractionation

Nuclear and cytoplasmic proteins were extracted using a Nuclear and Cytoplasmic Protein Extraction Kit (P0027, Beyotime, China) according to the manufacturer’s instructions and analyzed by western blotting. Lamin B and Tubulin served as nuclear and cytoplasmic markers, respectively.

### In vivo animal model

Male BALB/c nude mice (4–6 weeks old) were randomly assigned to experimental groups (*n* = 5 per group). 786-O cells stably expressing shNC or shSREBP1 (5 × 10^6^ cells suspended in 100 µL PBS/Matrigel, 1:1) were subcutaneously injected into the right flank of each mouse. Mice were euthanized 21 days after tumor implantation, and tumors were excised, photographed, weighed, fixed in 4% paraformaldehyde, paraffin-embedded, and processed for histological analyses.

Male BALB/c nude mice were subcutaneously injected with 5 × 10⁶ 786-O cells. Two weeks after tumor implantation, mice were randomly assigned to receive vehicle, SA (5 or 10 mg/kg), MK-6482 (1 mg/kg), or SA (10 mg/kg) combined with MK-6482 (1 mg/kg) by oral gavage twice daily. Tumor growth was monitored throughout the treatment period. After 15 days, tumors were harvested for histological, lipidomic, and molecular analyses. All animal experiments were approved by the Animal Ethics Committee of Hunan University of Chinese Medicine (Approval No. SLBH-202604210007).

### Statistical analysis

Data are presented as mean ± SD unless otherwise indicated. Statistical analyses were performed using GraphPad Prism 9. Comparisons between two groups were conducted using Student’s *t*-test, whereas multiple-group comparisons were analyzed using one-way or two-way ANOVA. A two-sided *P* < 0.05 was considered statistically significant.

## Results

### SREBP1 is required for lipid metabolic reprogramming and malignant phenotypes in ccRCC

**Fig. 1:**
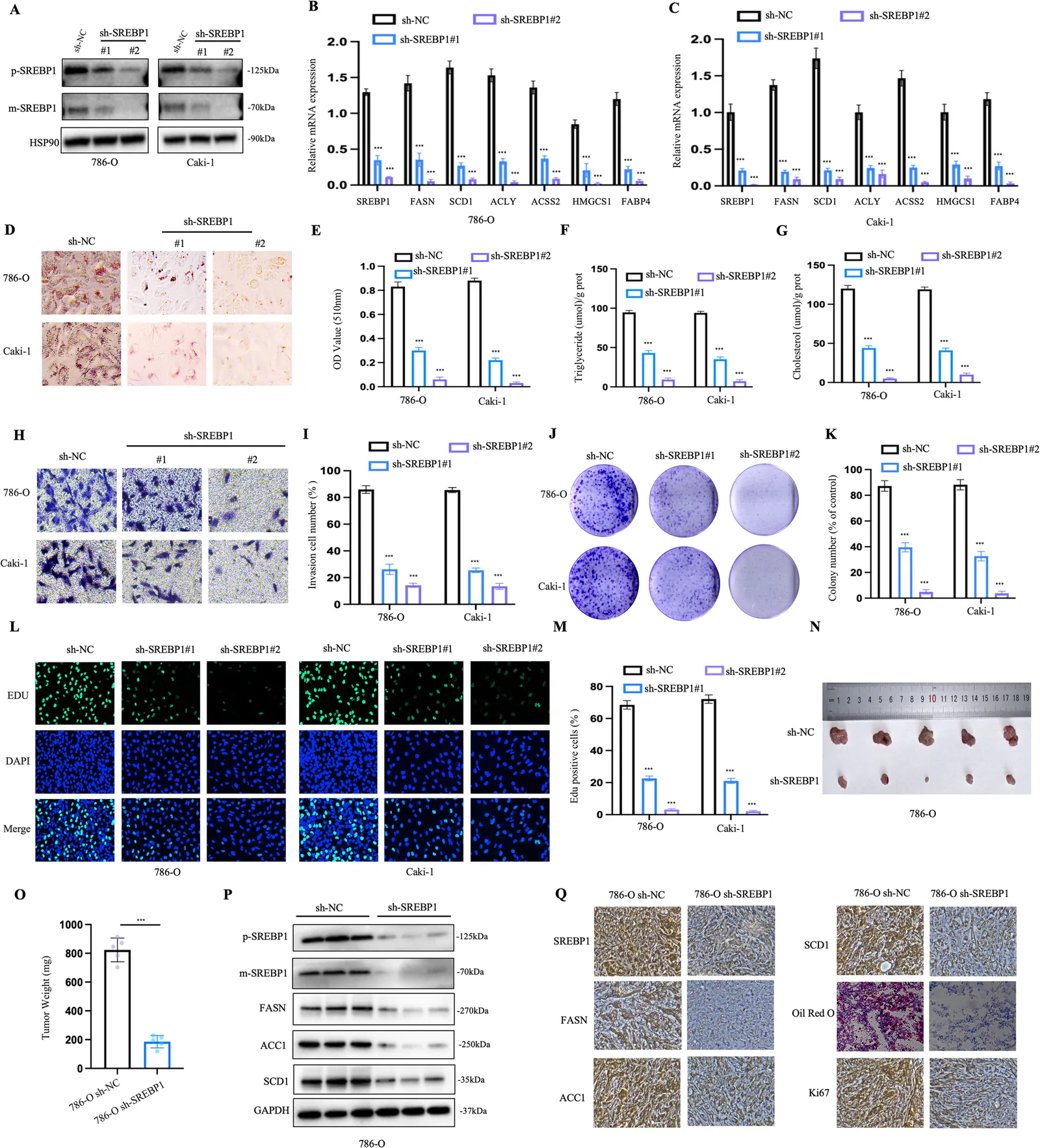
SREBP1 is required for lipid metabolic reprogramming and malignant phenotypes in ccRCC. **A** Immunoblot analysis of precursor SREBP1 (p-SREBP1) and mature SREBP1 (m-SREBP1) in 786-O and Caki-1 cells transduced with control shRNA (sh-NC) or two independent SREBP1 shRNAs; HSP90 served as a loading control. (B-C) RT-qPCR analysis of SREBP1 and lipid metabolism-related genes (FASN, SCD1, ACLY, ACSS2, HMGCS1, and FABP4) in 786-O (**B**) and Caki-1 (**C**) cells. **D**, **E** Representative Oil Red O staining images and quantitative analysis of intracellular lipid droplets. **F**, **G** Measurement of cellular triglyceride and total cholesterol levels following SREBP1 knockdown. **H**, **I** Transwell invasion assays and quantification of invaded cells. **J**, **K** Colony formation assays showing clonogenic capacity. **L**, **M** EdU incorporation assays and quantification of proliferating cells (scale bar, 100 μm). **N** Representative images of xenograft tumors derived from 786-O shNC or shSREBP1 cells (n = 5 mice per group). **O** Tumor weights of xenografts at the experimental endpoint. **P** Immunoblot analysis of p-SREBP1, m-SREBP1, FASN, ACC1, and SCD1 in xenograft tumors. GAPDH served as the loading control. **Q** Representative Oil Red O staining and immunohistochemical staining for SREBP1, FASN, ACC1, SCD1, and Ki67 in xenograft tumors. Data are presented as mean ± SD from three independent biological experiments. Statistical significance was determined using one-way ANOVA. *P < 0.05, **P < 0.01, ***P < 0.001

SREBP1 is a master regulator of de novo lipogenesis and is frequently activated in multiple malignancies. To determine its functional role in ccRCC, SREBP1 was stably silenced in 786-O and Caki-1 cells. Knockdown of SREBP1 markedly decreased the abundance of mature SREBP1 (Fig. 1A). Expression of multiple lipid metabolism-related genes was also substantially reduced following SREBP1 depletion (Fig. 1B, C).

Given the central role of SREBP1 in lipid biosynthesis, we next examined intracellular lipid accumulation. Oil Red O staining revealed a pronounced reduction in lipid droplet content in SREBP1-depleted cells compared with control cells (Fig. 1D), which was further confirmed by quantitative measurement of Oil Red O absorbance (Fig. 1E). Biochemical assays showed that cellular triglyceride and cholesterol levels were significantly decreased following SREBP1 knockdown in both ccRCC cell lines (Fig. 1F, G), indicating impaired lipid metabolic reprogramming. We next evaluated whether SREBP1 depletion affected malignant cellular behaviors. Transwell invasion assays demonstrated that suppression of SREBP1 markedly reduced the invasive capacity of 786-O and Caki-1 cells (Fig. 1H, I). Furthermore, colony formation assays revealed that SREBP1 knockdown significantly impaired long-term clonogenic growth in both cell lines (Fig. 1J, K). EdU incorporation assays further demonstrated a marked reduction in proliferating cells following SREBP1 depletion (Fig. 1L, M).

To validate these findings in vivo, we established subcutaneous xenograft models using 786-O cells stably expressing control shRNA or shSREBP1. Genetic depletion of SREBP1 markedly suppressed tumor growth, as evidenced by the reduced tumor size and tumor weight in the shSREBP1 group (Fig. 1N, O). Immunoblotting of xenograft tissues confirmed efficient SREBP1 knockdown and concomitant downregulation of FASN, ACC1, and SCD1 (Fig. 1P). Histological examination revealed markedly reduced lipid accumulation together with decreased Ki67, FASN, ACC1, and SCD1 staining in shSREBP1 tumors (Fig. 1Q). Quantitative analysis of immunohistochemical staining and Oil Red O-positive area further confirmed these observations (Supplementary Fig. S1). Collectively, these findings establish SREBP1 as a key regulator of lipid metabolic reprogramming and malignant progression in ccRCC.

### SA suppresses SREBP1-dependent lipogenic signaling in ccRCC cells

To identify small-molecule regulators of SREBP1 signaling, we performed high-throughput screening of a natural product compound library comprising approximately 4,000 compounds. Immunofluorescence-based screening identified Soulangianolide A (SA) as a candidate molecule that markedly reduced nuclear accumulation of SREBP1 and induced apoptotic morphology in ccRCC cells, suggesting inhibition of SREBP1-dependent transcriptional activity (Supplementary Fig. S2A). Because of its limited natural abundance, SA was chemically synthesized (Supplementary Fig. S2B) and subsequently evaluated for its effects on lipid metabolic reprogramming and malignant phenotypes.

**Fig. 2:**
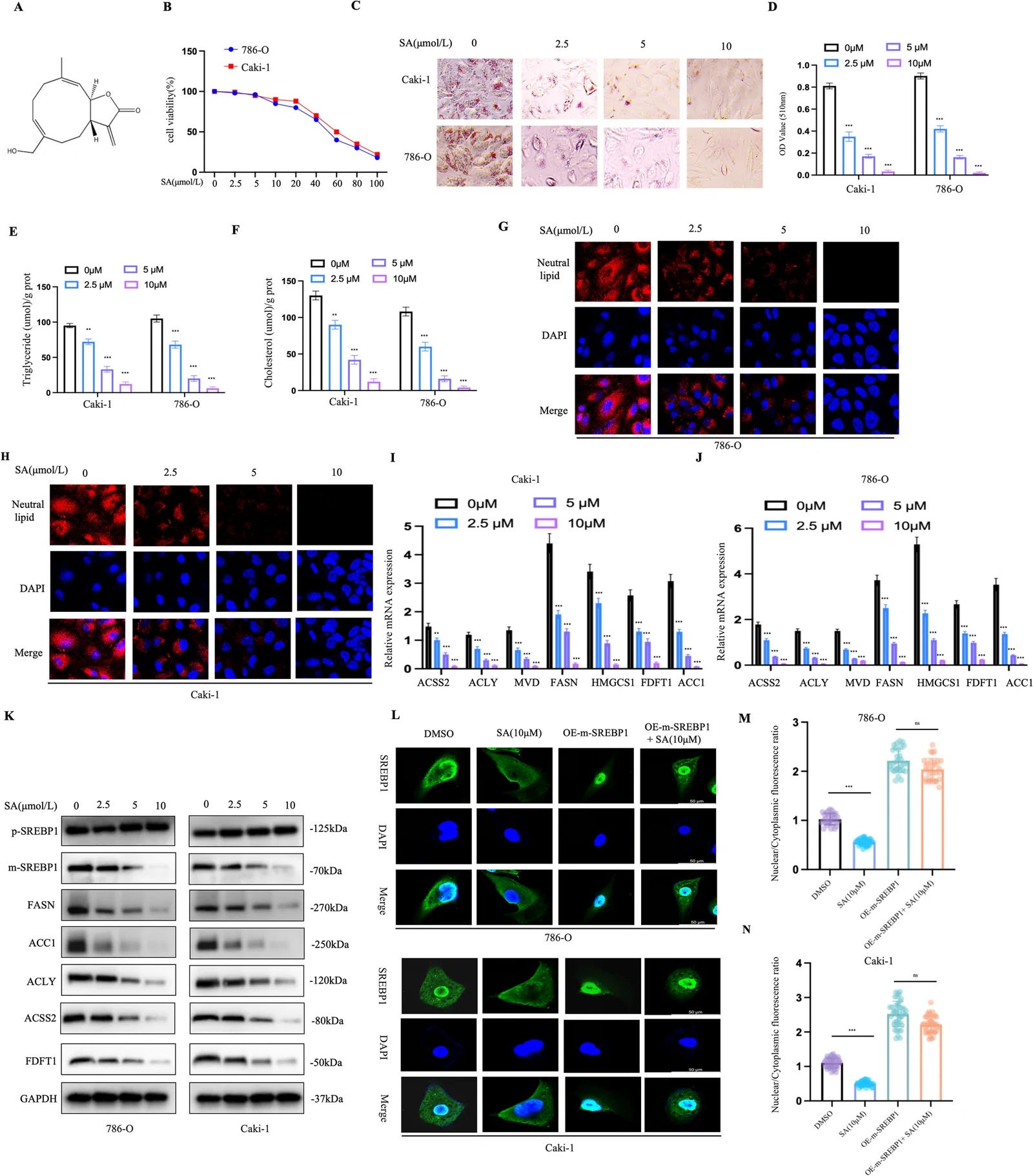
SA suppresses SREBP1-dependent lipogenic signaling in ccRCC cells. **A** Chemical structure of SA. **B** Cell viability of 786-O and Caki-1 cells treated with increasing concentrations of SA, assessed by CCK-8 assay. **C**, **D** Oil Red O staining images and quantitative analysis of intracellular lipid accumulation. **E**, **F** Intracellular triglyceride and total cholesterol levels in ccRCC cells following SA treatment. **G**, **H** Fluorescence images of neutral lipid staining (red) and DAPI nuclear staining (blue; scale bar, 100 μm). **I**, **J** RT-qPCR analysis of lipid metabolism related gene expression in Caki-1 (**I**) and 786-O (**J**) cells after SA treatment. **K** Immunoblot analysis of precursor and mature SREBP1, and lipid metabolism-related proteins in SA-treated cells; GAPDH served as a loading control. **L**-**N** Immunofluorescence analysis and quantification of SREBP1 nuclear localization in cells expressing constitutively active mature SREBP1 (OE-mSREBP1) with or without SA treatment (blue; scale bar, 50 μm), together with quantification of the nuclear/cytoplasmic fluorescence ratio. Data are presented as mean ± SD from three independent biological experiments. Statistical significance was determined using one-way ANOVA. *P < 0.05, **P < 0.01, ***P < 0.001

The chemical structure of SA is shown in Fig. 2A. CCK-8 assays demonstrated that SA treatment reduced the viability of 786-O and Caki-1 cells in a dose-dependent manner (Fig. 2B). Oil Red O staining revealed a marked reduction in intracellular lipid droplets following SA treatment, with progressively diminished lipid accumulation at increasing concentrations (Fig. 2C, D). Intracellular triglyceride and total cholesterol contents were significantly reduced following SA exposure (Fig. 2E, F). Neutral lipid staining further demonstrated markedly diminished lipid droplet formation accompanied by reduced fluorescence intensity, indicating broad suppression of intracellular lipid storage (Fig. 2G, H). RT-qPCR analysis showed that SA dose-dependently downregulated multiple genes involved in fatty acid and cholesterol biosynthesis in both 786-O and Caki-1 cells (Fig. 2I, J). Western blot analysis revealed that SA treatment resulted in a dose-dependent reduction in the mature form of SREBP1, accompanied by decreased expression of downstream lipogenic enzymes including FASN, ACC1, ACLY, ACSS2, and FDFT1, suggesting inhibition of SREBP1-driven lipogenic signaling (Fig. 2K). Immunofluorescence analysis further demonstrated that SA markedly attenuated nuclear accumulation of SREBP1. In contrast, ectopic expression of constitutively active mature SREBP1 (OE-mSREBP1) partially restored nuclear SREBP1 signaling activity in the presence of SA, as reflected by an increased nuclear-to-cytoplasmic fluorescence ratio (Fig. 2L-N).

These findings demonstrate that SA suppresses SREBP1-dependent lipogenic signaling, leading to impaired lipid metabolic reprogramming, reduced lipid accumulation, and suppression of malignant phenotypes.

### SA induces global lipidomic remodeling toward a lipotoxic state

**Fig. 3:**
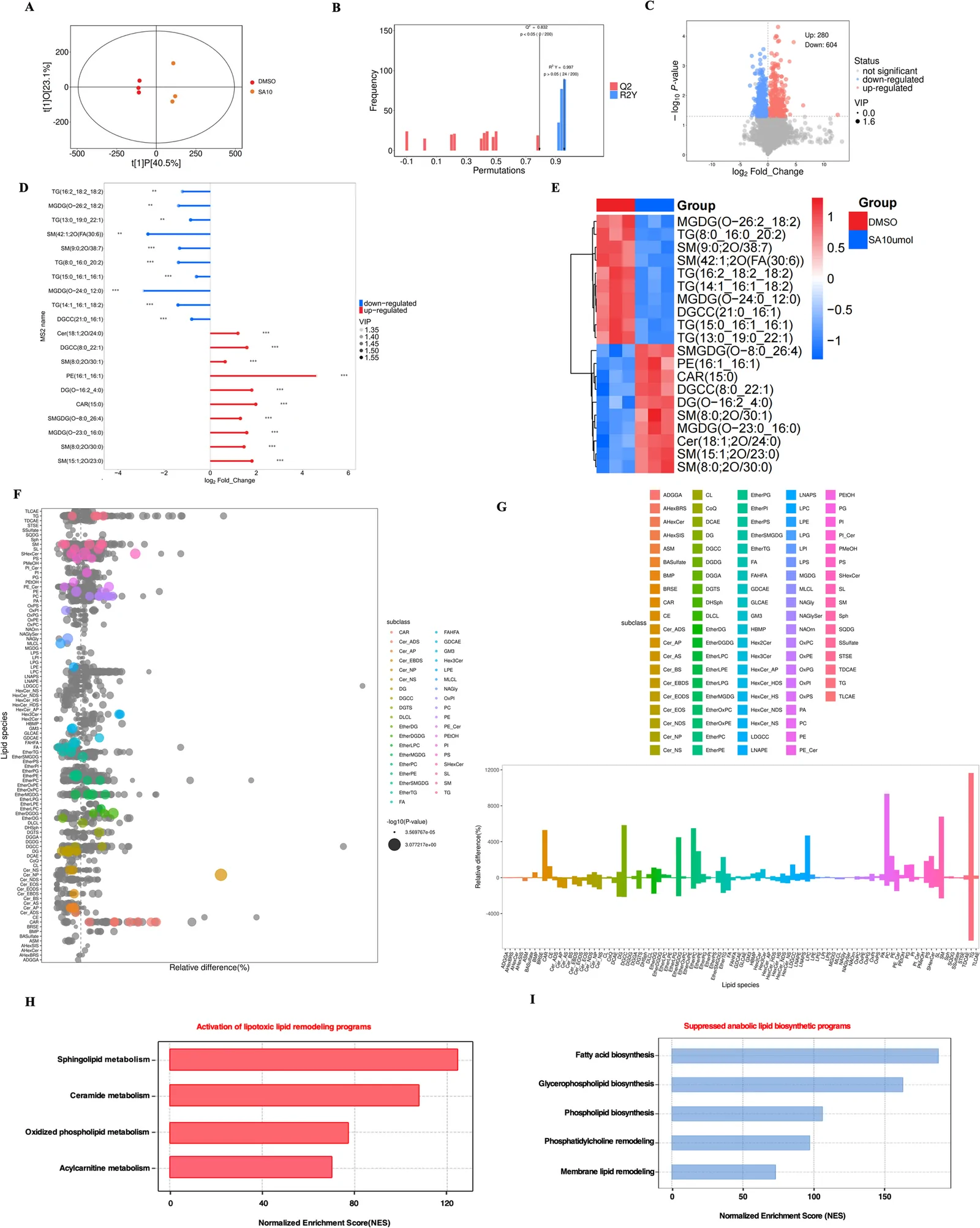
SA induces global lipidomic remodeling toward a lipotoxic state. **A** Principal component analysis (PCA) of lipidomic profiles in DMSO- and SA-treated ccRCC cells. **B** OPLS-DA permutation test validating the lipidomic model. **C** Volcano plot of differentially abundant lipid species after SA treatment. **D** Representative differential lipid species ranked by log₂ fold change and VIP score. **E** Hierarchical clustering heatmap of the top differentially abundant lipid species. **F** Bubble plot showing changes in lipid subclasses after SA treatment. **G** Relative differences of lipid subclasses between DMSO- and SA-treated cells. **H** Enrichment analysis of activated lipotoxic lipid remodeling pathways. **I** Enrichment analysis of suppressed anabolic lipid biosynthetic pathways

To comprehensively characterize SA-induced metabolic alterations, untargeted lipidomic profiling was performed in ccRCC cells treated with SA or DMSO. Principal component analysis (PCA) demonstrated a clear separation between control and SA-treated samples, indicating that SA reshaped the overall lipidomic landscape of ccRCC cells (Fig. 3A). The robustness and predictive performance of the OPLS-DA model were further validated by permutation testing, which showed high R²Y and Q² values without evidence of overfitting (Fig. 3B).

Volcano plot analysis identified widespread lipid alterations following SA treatment, including 280 significantly upregulated and 604 significantly downregulated lipid species, revealing extensive remodeling of lipid metabolism (Fig. 3C). Differential lipid analysis further revealed that multiple triglyceride, monogalactosyldiacylglycerol, and sphingomyelin species were markedly decreased, whereas ceramide, acylcarnitine, phosphatidylethanolamine, and diacylglycerol-containing lipids were significantly increased (Fig. 3D). Hierarchical clustering of the top differential lipid species further demonstrated distinct lipidomic signatures between DMSO- and SA-treated cells, indicating coordinated lipid remodeling induced by SA (Fig. 3E). The complete hierarchical clustering heatmap of all differentially abundant lipid metabolites (|log₂FC| > 1) is provided in Additional file one.

To further evaluate global lipid remodeling, lipid subclass analysis demonstrated extensive alterations across multiple lipid categories following SA treatment (Fig. 3F, G). Storage-associated neutral lipids, including triglycerides and several membrane lipid subclasses, exhibited an overall decreasing trend, whereas ceramide and acylcarnitine-related lipid subclasses showed marked accumulation, indicating a shift toward a lipotoxic lipid profile.

Pathway enrichment analysis further revealed that SA substantially reprogrammed lipid metabolism. Lipotoxic lipid remodeling pathways, including sphingolipid metabolism, ceramide metabolism, oxidized phospholipid metabolism, and acylcarnitine metabolism, were significantly enriched (Fig. 3H). In contrast, anabolic lipid biosynthetic pathways, including fatty acid biosynthesis, glycerophospholipid biosynthesis, phospholipid biosynthesis, phosphatidylcholine remodeling, and membrane lipid remodeling, were markedly suppressed (Fig. 3I). These findings demonstrate that SA induces extensive lipidomic remodeling by shifting ccRCC cells from an anabolic lipogenic state toward a lipotoxic metabolic state, which is consistent with the inhibition of SREBP1-driven lipogenic signaling.

### SA directly targets FAM120A and disrupts its interaction with SREBP1

**Fig. 4:**
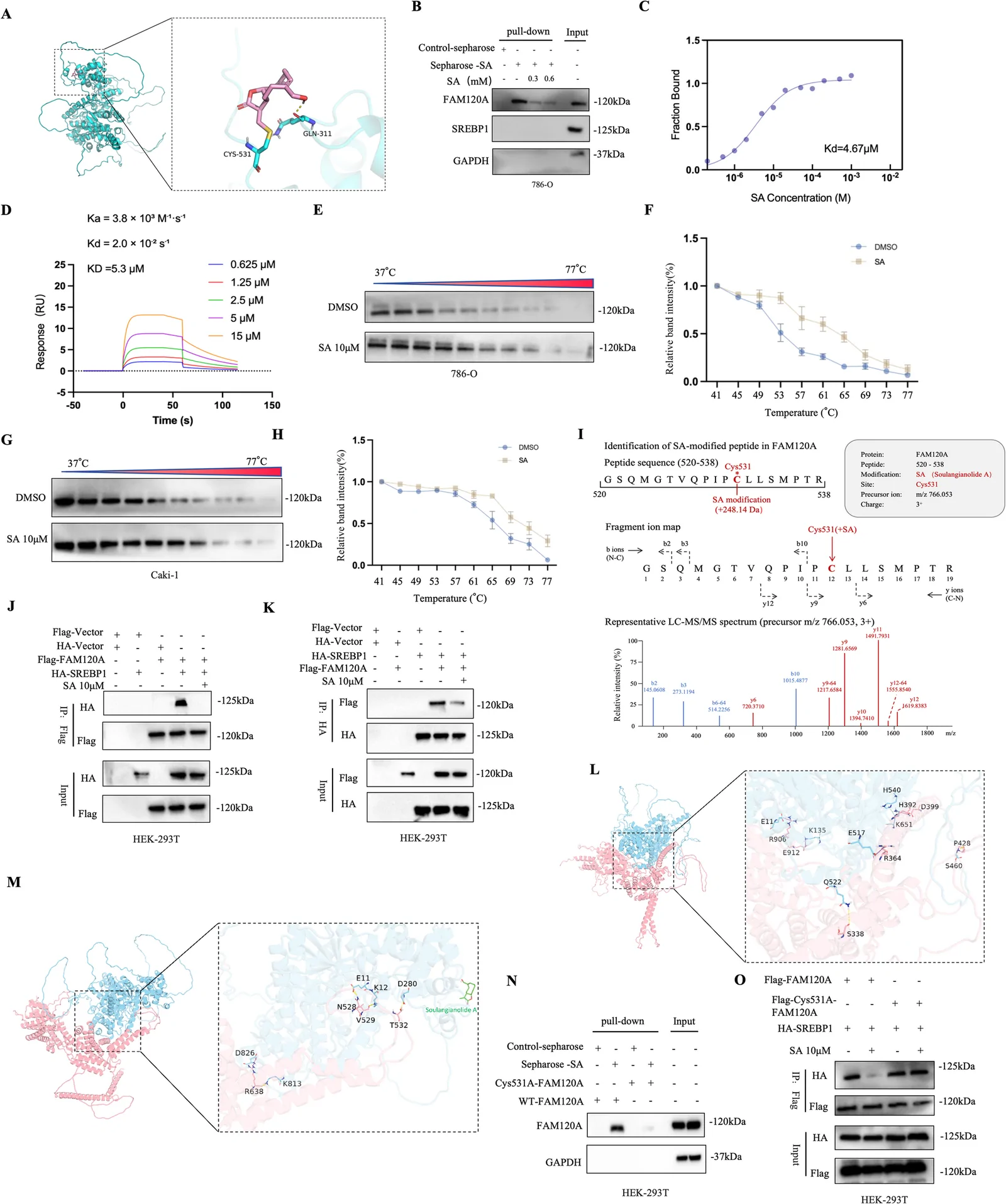
SA directly targets FAM120A and disrupts its interaction with SREBP1. **A** Molecular docking model showing the predicted binding mode of SA within the FAM120A binding pocket. **B** Pull-down assay using SA-conjugated sepharose beads followed by immunoblotting to detect FAM120A and SREBP1 in 786-O cells. **C**, **D** SPR analysis showing the dose-response curve (**C**) and binding kinetics (**D**) between SA and recombinant FAM120A. **E**-**H** CETSA analysis and quantification of FAM120A thermal stability in 786-O and Caki-1 cells treated with DMSO or SA. **I** Representative LC-MS/MS spectrum identifying the peptide containing the SA-modified Cys531 residue of FAM120A. **J**, **K** Co-immunoprecipitation assays showing the interaction between FAM120A and SREBP1 in HEK-293T cells and its disruption by SA treatment. **L** Structural model of the FAM120A-SREBP1 complex. **M** Structural model of the FAM120A-SA-SREBP1 complex showing reduced intermolecular interactions after SA binding. **N** Pull-down assay comparing wild-type FAM120A and the C531A mutant. **O** Co-immunoprecipitation analysis showing that the C531A mutation abolishes SA-mediated disruption of the FAM120A-SREBP1 interaction. Data are presented as mean ± SD from three independent biological experiments

Given the emerging role of FAM120A in lipid metabolism, we first examined its interaction with lipid metabolism-related transcription factors. Co-immunoprecipitation analyses demonstrated that FAM120A preferentially interacted with mature SREBP1 compared with other transcription factors, including SREBP2, ChREBP, PPARγ, and p300 (Supplementary Fig. S3A). Moreover, SA selectively disrupted the interaction between FAM120A and SREBP1 without broadly affecting its association with other proteins involved in lipid metabolism or oncogenic signaling, including RNA-binding proteins and components of the transcriptional machinery (Supplementary Fig. S3B-C). We next investigated whether FAM120A serves as a direct molecular target of SA. Molecular docking analysis predicted that SA occupies a defined binding pocket within FAM120A and forms stable interactions with Cys531 and Gln311 (Fig. 4A).

To experimentally validate this interaction, SA-conjugated Sepharose pull-down assays specifically enriched FAM120A but not SREBP1 from 786-O cell lysates, indicating direct binding of SA to FAM120A rather than to SREBP1 (Fig. 4B). SPR analysis further confirmed a direct interaction between SA and recombinant FAM120A protein, with an equilibrium dissociation constant (KD) in the low micromolar range (Fig. 4C, D), indicating a specific binding affinity. In addition, CETSA demonstrated that SA treatment increased the thermal stability of FAM120A in both 786-O and Caki-1 cells (Fig. 4E-H), providing additional evidence for direct target engagement in cells. To identify the SA-binding residue, LC-MS/MS analysis was performed following incubation of recombinant FAM120A with SA. A modified peptide containing Cys531 was identified with high-confidence fragment ion assignment, demonstrating covalent modification of this residue by SA (Fig. 4I).

Given that FAM120A has been reported to form a functional complex with SREBP1 [17], we next examined whether SA influences this interaction. Co-immunoprecipitation assays demonstrated a robust interaction between FAM120A and SREBP1 in HEK-293T cells, which was attenuated upon SA treatment (Fig. 4J, K), indicating that SA disrupts the FAM120A-SREBP1 complex.

Structural modeling further revealed extensive hydrogen bonds, π–π stacking interactions, and salt bridges at the FAM120A-SREBP1 interface, whereas incorporation of SA markedly reduced these intermolecular interactions, suggesting that SA binding destabilizes the protein complex (Fig. 4L, M). To further validate the predicted binding site, a Cys531Ala (C531A) mutant of FAM120A was generated. SA-sepharose pull-down assays showed substantially reduced binding of the C531A mutant compared with wild-type FAM120A (Fig. 4N). Moreover, SA disrupted the interaction between wild-type FAM120A and SREBP1, whereas the C531A mutation largely abolished this inhibitory effect (Fig. 4O). These findings demonstrate that SA directly targets FAM120A through Cys531, thereby disrupting the FAM120A-SREBP1 complex and suppressing SREBP1-dependent lipogenic signaling.

### Cys531 of FAM120A is required for SA-mediated inhibition of SREBP1-dependent lipogenic signaling and malignant phenotypes in ccRCC cells

**Fig. 5:**
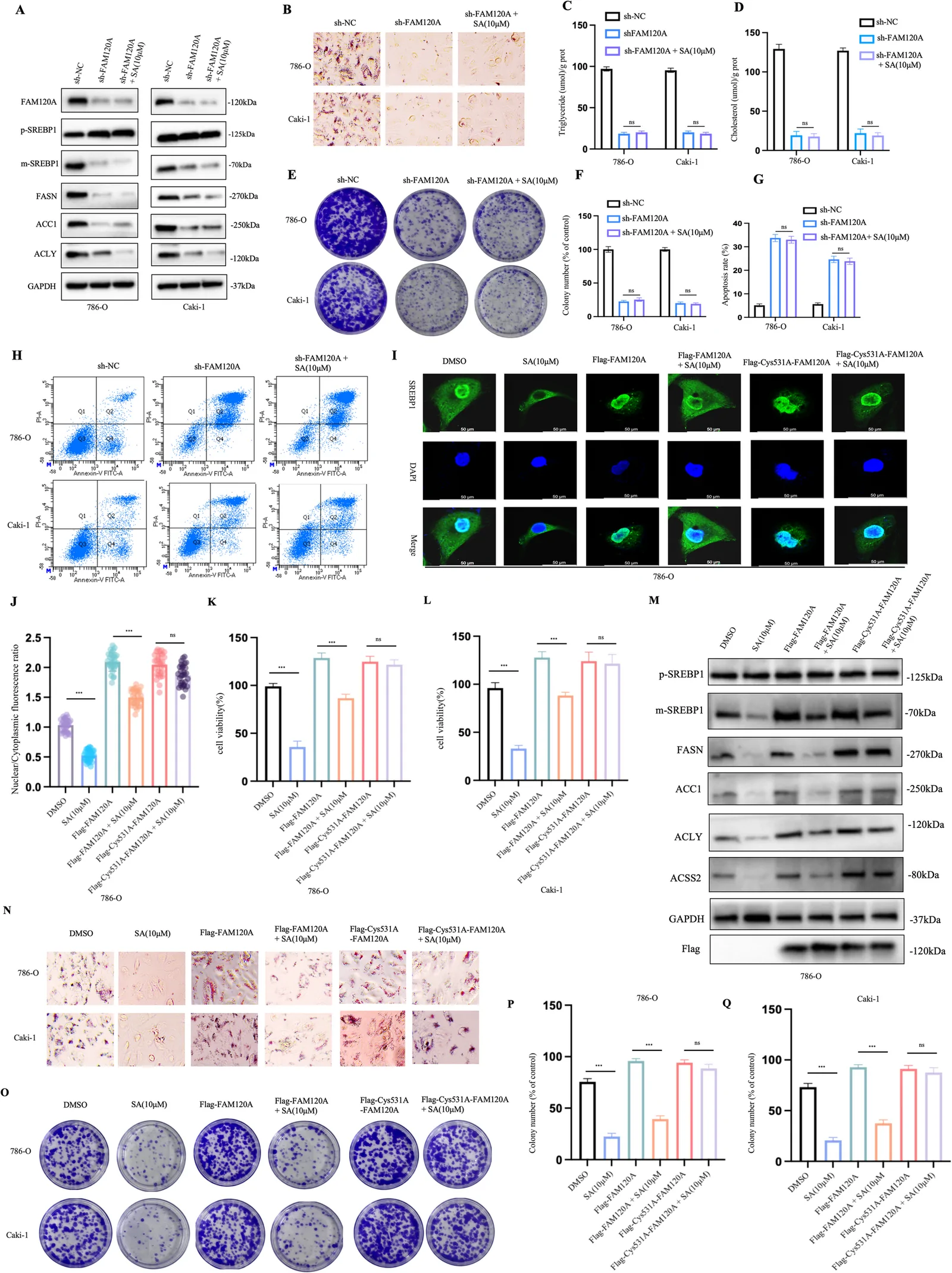
Cys531 of FAM120A is required for SA-mediated inhibition of SREBP1-dependent lipogenic signaling and malignant phenotypes in ccRCC cells. **A** Immunoblot analysis of FAM120A, precursor and mature SREBP1, and downstream lipogenic proteins in sh-NC-, sh-FAM120A-, or sh-FAM120A + SA-treated 786-O and Caki-1 cells. **B** Representative Oil Red O staining images showing intracellular lipid accumulation. **C**, **D** Quantification of intracellular triglyceride (**C**) and total cholesterol (**D**) levels. **E**, **F** Colony formation assays and quantification of colony numbers. **G**, **H** Annexin V-FITC/PI flow cytometry plots and quantification of apoptotic cells. **I**, **J** Immunofluorescence analysis and quantification of SREBP1 nuclear localization in cells expressing WT-FAM120A or C531A-FAM120A with or without SA treatment (scale bars,50 μm). **K**, **L** Cell viability analysis of 786-O and Caki-1 cells under the indicated treatments. **M** Immunoblot analysis of precursor SREBP1, mature SREBP1, and downstream lipogenic proteins in cells expressing WT-FAM120A or C531A-FAM120A in the presence or absence of SA. **N** Representative Oil Red O staining images under the indicated treatments. **O**-**Q** Colony formation assays and quantification of colony numbers in 786-O and Caki-1 cells. Data are presented as mean ± SD from three independent biological experiments

To further define the role of FAM120A in SREBP1-dependent lipogenic signaling, we next examined whether FAM120A regulates SREBP1-dependent transcription. ChIP-qPCR and ChIP-PCR analyses demonstrated that FAM120A depletion markedly reduced SREBP1 occupancy at the promoters of its target genes, including FASN, ACC1, and SCD1, indicating that FAM120A promotes SREBP1-dependent lipogenic transcription (Supplementary Fig. S3D-F). Consistently, ectopic expression of mature SREBP1 partially restored the expression of downstream lipogenic proteins suppressed by FAM120A knockdown (Supplementary Fig. S3G). Given the essential role of FAM120A in lipogenic signaling, we next investigated whether the metabolic effects of SA depend on FAM120A. Silencing FAM120A reduced the abundance of mature SREBP1 together with its downstream lipogenic enzymes, including FASN, ACC1, ACLY, and ACSS2, in both 786-O and Caki-1 cells (Fig. 5A). Importantly, SA treatment did not produce additional inhibitory effects on these proteins in FAM120A-depleted cells, indicating that inhibition of SREBP1 signaling by SA requires FAM120A.

Consistent with these findings, Oil Red O staining revealed that depletion of FAM120A significantly reduced intracellular lipid droplet accumulation, whereas additional SA treatment did not produce further reductions (Fig. 5B). Intracellular triglyceride and total cholesterol levels were similarly decreased by FAM120A knockdown and remained unchanged following SA treatment (Fig. 5C, D), indicating that the lipid metabolic effects of SA are largely FAM120A dependent.

We next examined whether FAM120A is required for the antitumor activity of SA. Colony formation assays showed that FAM120A depletion markedly impaired clonogenic growth in both ccRCC cell lines, while subsequent SA treatment produced no additional inhibitory effects (Fig. 5E, F). Flow cytometric analysis further demonstrated that silencing FAM120A significantly increased apoptotic cell death, which was not further enhanced by SA treatment (Fig. 5G, H). These findings indicate that both the metabolic and antitumor activities of SA require FAM120A. To further validate the functional importance of the SA-binding residue, wild-type FAM120A (WT-FAM120A) and the C531A mutant were ectopically expressed in ccRCC cells. Immunofluorescence analysis demonstrated that SA reduced nuclear accumulation of SREBP1, whereas WT-FAM120A partially restored SREBP1 nuclear localization. In contrast, expression of C531A-FAM120A largely abolished the inhibitory effect of SA, resulting in persistent nuclear localization of SREBP1 despite SA treatment (Fig. 5I, J).

Consistently, SA significantly reduced cell viability in both 786-O and Caki-1 cells, whereas WT-FAM120A overexpression partially rescued this inhibitory effect. Notably, expression of C531A-FAM120A conferred substantially greater resistance to SA-mediated growth inhibition than WT-FAM120A (Fig. 5K, L). Western blot analysis further demonstrated that SA effectively suppressed mature SREBP1 and downstream lipogenic proteins in WT-FAM120A-expressing cells, whereas these inhibitory effects were largely abolished in cells expressing C531A-FAM120A (Fig. 5M). Similarly, SA markedly reduced intracellular lipid accumulation and colony-forming ability in WT-FAM120A-expressing cells, while C531A-FAM120A significantly attenuated these effects (Fig. 5N-Q). Consistent with its effects on lipid metabolism and SREBP1 signaling, Cys531 mutation also attenuated SA-mediated inhibition of cell invasion and proliferation, as demonstrated by Transwell and EdU assays (Supplementary Fig. S4A-F).

Consistent with the central role of SREBP1, ectopic expression of mature SREBP1 partially restored lipogenic protein expression, lipid accumulation, colony formation, and cell viability in SA-treated or FAM120A-depleted cells (Supplementary Fig. S5A-J), further supporting SREBP1 as a major downstream effector of FAM120A. These findings demonstrate that Cys531 is required for SA-mediated inhibition of FAM120A-SREBP1 signaling, lipid metabolic reprogramming, and malignant phenotypes in ccRCC cells.

### SA suppresses PPARγ signaling through inhibition of SREBP1-dependent lipogenic signaling

**Fig. 6:**
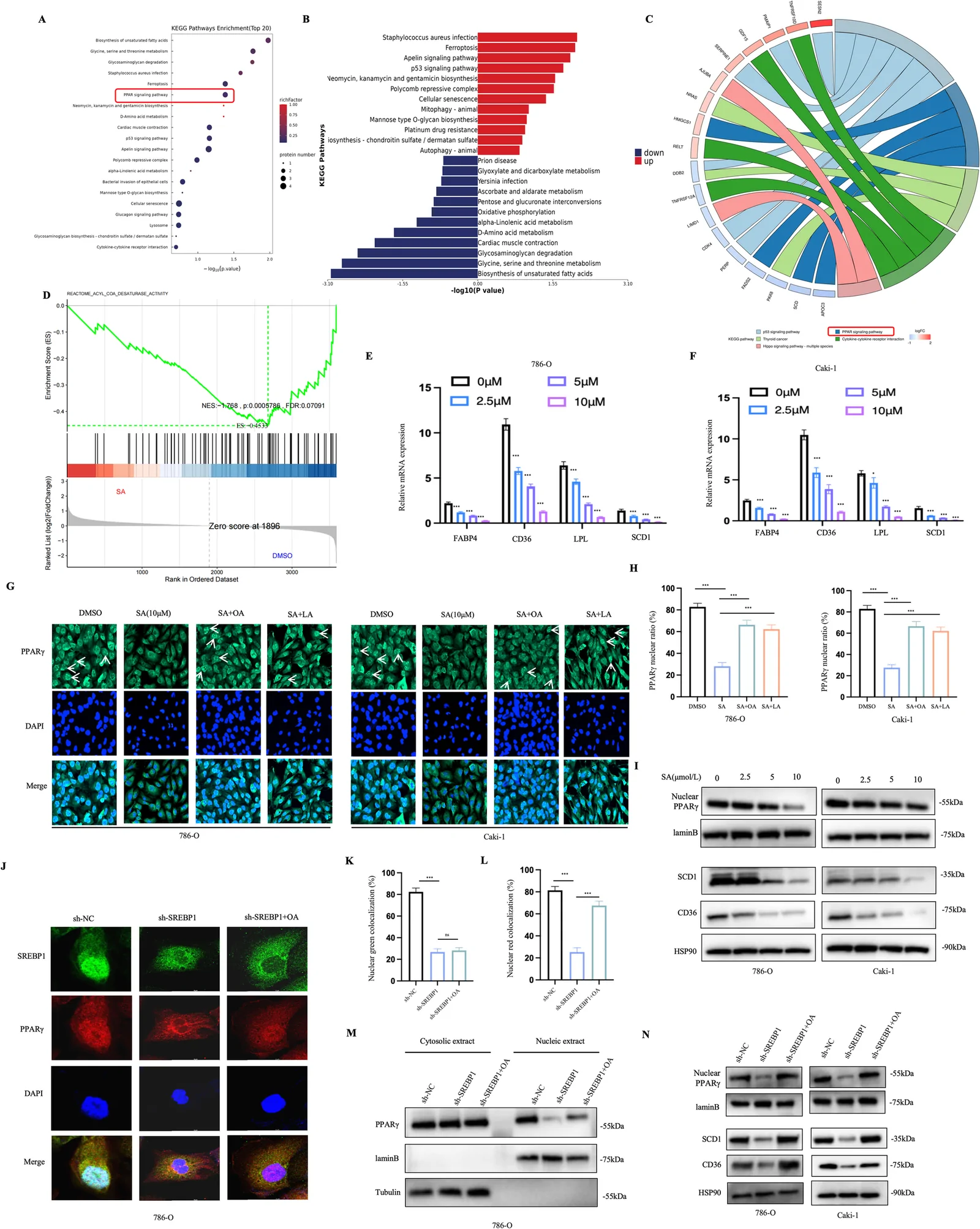
SA suppresses PPARγ signaling via lipid-dependent metabolic regulation. **A** KEGG pathway enrichment analysis highlighting lipid metabolism-related pathways in SA-treated cells. **B** KEGG functional classification of differentially expressed proteins. **C** Chord diagram showing associations between key genes and enriched lipid-related pathways. **D** GSEA of acyl-CoA desaturase activity-associated gene sets. **E**, **F** Relative mRNA expression of PPARγ target genes (FABP4, CD36, LPL, and SCD1) in 786-O and Caki-1 cells treated with increasing concentrations of SA. **G**, **H** Representative immunofluorescence images and quantification of PPARγ nuclear localization in 786-O and Caki-1 cells treated with SA alone or in combination with OA or LA. DAPI was used for nuclear staining (scale bars,100 μm). **I** Immunoblot analysis of nuclear PPARγ and the downstream PPARγ target proteins SCD1 and CD36 in 786-O and Caki-1 cells treated with increasing concentrations of SA. Lamin B and HSP90 served as nuclear and loading controls, respectively. **J** Representative immunofluorescence images of SREBP1 (green) and PPARγ (red) in 786-O cells under the indicated conditions (scale bars,50 μm). **K** Quantification of SREBP1 nuclear localization. **L** Quantification of PPARγ nuclear localization. **M** Nuclear-cytoplasmic fractionation analysis of PPARγ in 786-O cells. Lamin B and Tubulin served as nuclear and cytoplasmic markers, respectively. **N** Immunoblot analysis of nuclear PPARγ, SCD1, and CD36 in 786-O and Caki-1 cells under the indicated conditions. Lamin B and HSP90 served as nuclear and loading controls, respectively. Data are presented as mean ± SD from three independent biological experiments. *P < 0.05, **P < 0.01, ***P < 0.001

To further characterize the molecular mechanisms underlying SA-mediated lipid metabolic reprogramming, quantitative proteomic profiling was performed in ccRCC cells. KEGG pathway enrichment analysis revealed that SA predominantly affected lipid metabolic pathways, including PPAR signaling, biosynthesis of unsaturated fatty acids, α-linolenic acid metabolism, and oxidative phosphorylation (Fig. 6A). Pathway directionality analysis further demonstrated suppression of multiple metabolic pathways accompanied by activation of stress-related pathways, including ferroptosis, p53 signaling, and autophagy (Fig. 6B). Consistently, gene-pathway interaction analysis identified coordinated downregulation of multiple PPAR signaling components, including SCD1, FADS2, and APOC3 (Fig. 6C). GSEA further revealed significant negative enrichment of the Reactome acyl-CoA desaturase activity gene set following SA treatment (Fig. 6D), indicating global inhibition of fatty acid desaturation pathways. In addition, independent transcriptomic analysis further demonstrated coordinated suppression of metabolic genes together with activation of stress-responsive programs following SA treatment (Supplementary Fig. S6A-D), consistent with inhibition of FAM120A-SREBP1 dependent lipogenic transcription and subsequent attenuation of lipid-dependent PPARγ signaling.

Given the central role of PPARγ in lipid uptake and storage [23–25], we next investigated whether SA regulates PPARγ signaling. RT-qPCR analysis demonstrated dose-dependent downregulation of canonical PPARγ target genes, including FABP4, CD36, LPL, and SCD1, in both 786-O and Caki-1 cells (Fig. 6E, F). Immunofluorescence analysis showed that SA markedly reduced nuclear accumulation of PPARγ, whereas supplementation with oleic acid (OA) or linoleic acid (LA) partially restored its nuclear localization (Fig. 6G, H). Immunoblot analysis of nuclear extracts demonstrated a dose-dependent reduction in nuclear PPARγ following SA treatment, accompanied by decreased expression of the downstream PPARγ target proteins SCD1 and CD36 (Fig. 6I), indicating suppression of PPARγ signaling.

To determine whether PPARγ inhibition results from impaired SREBP1-dependent lipogenesis, SREBP1 was silenced and cells were supplemented with exogenous OA. SREBP1 depletion markedly reduced the nuclear localization of both SREBP1 and PPARγ. Although OA supplementation failed to restore SREBP1 nuclear localization, it significantly rescued nuclear accumulation of PPARγ (Fig. 6J-L). Nuclear-cytoplasmic fractionation further confirmed recovery of nuclear PPARγ by OA supplementation (Fig. 6M). Consistently, OA partially restored the expression of nuclear PPARγ and its downstream target proteins SCD1 and CD36 in SREBP1-depleted cells (Fig. 6N), indicating that endogenous monounsaturated fatty acids generated through SREBP1-dependent lipogenesis are required for efficient PPARγ activation.

### SA suppresses tumor growth and lipogenic programs in vivo

To further validate the antitumor efficacy and mechanistic relevance of SA in vivo, a xenograft mouse model was established. MK-6482 (belzutifan) is a selective HIF-2α inhibitor that has been developed as a therapeutic option for ccRCC [26–29]. Because the HIF-2α downstream effector MED15 promotes ccRCC progression through activation of SREBP-dependent lipogenesis [30], we evaluated SA alone or in combination with MK-6482 to determine whether targeting lipid metabolic reprogramming could enhance therapeutic efficacy.

**Fig. 7:**
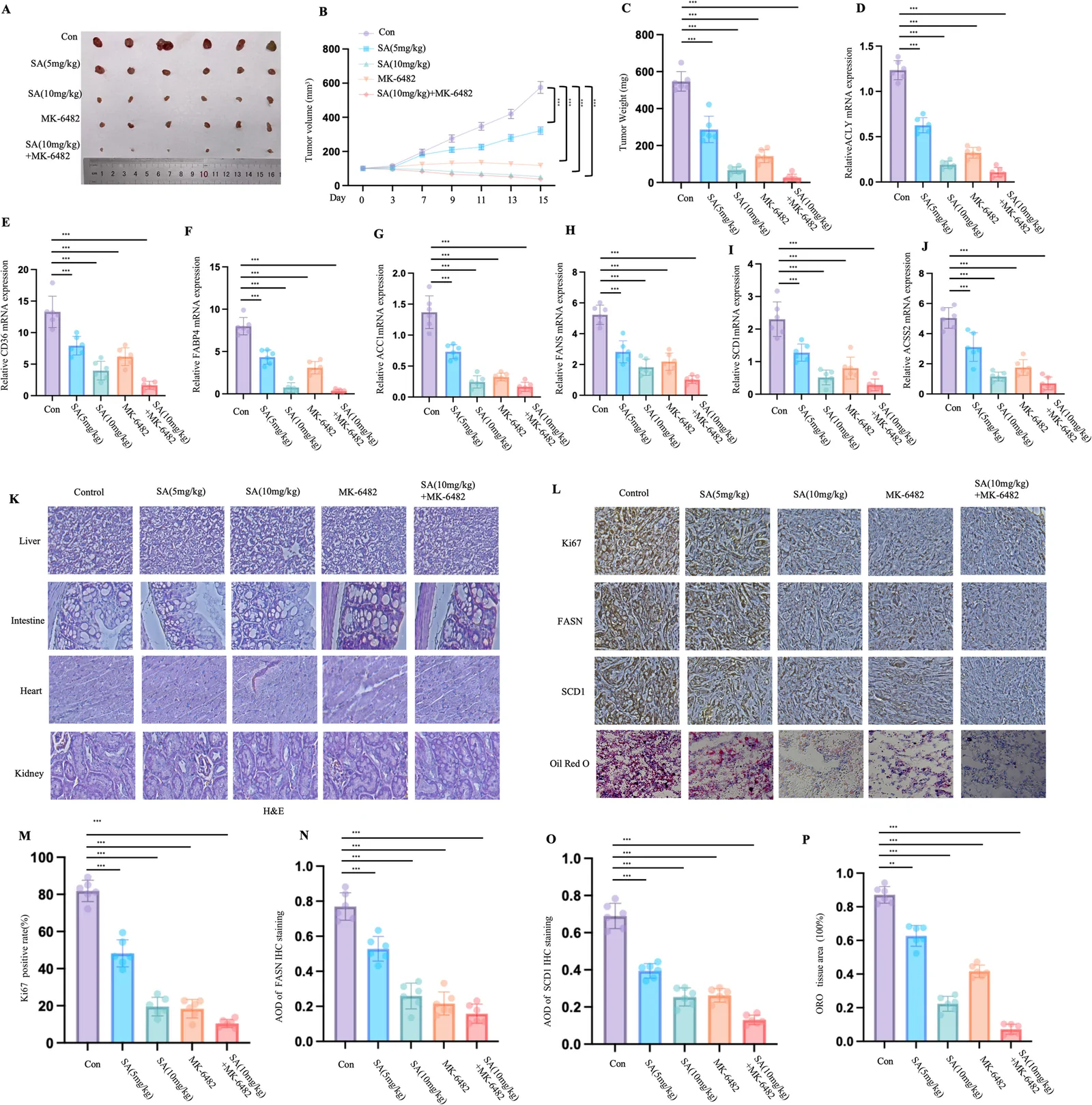
SA suppresses tumor growth and lipogenic programs in vivo. **A** Representative images of xenograft tumors from the indicated treatment groups. **B** Tumor growth curves during the treatment period. **C** Tumor weights at the experimental endpoint. **D-J** RT-qPCR analysis of lipid metabolism-related genes in xenograft tumors, including ACLY (**D**), CD36 (**E**), FABP4 (**F**), ACC1 (**G**), FASN (**H**), SCD1 (**I**), and ACSS2 (**J**). **K** Representative H&E staining of major organs (liver, intestine, heart, and kidney) showing no apparent histopathological abnormalities (scale bar, 100 μm). **L** Representative immunohistochemical staining for Ki67, FASN, and SCD1, together with Oil Red O staining of xenograft tumors (scale bar, 100 μm). **M** Quantification of Ki67-positive cells. **N**, **O** Quantification of FASN and SCD1 staining by average optical density (AOD). **P** Quantification of Oil Red O-positive lipid area. Data are presented as mean ± SD (n = 6 mice per group). Statistical significance was determined by one-way ANOVA. *P < 0.05, **P < 0.01, ***P < 0.001

SA treatment significantly suppressed xenograft growth in a dose-dependent manner, with the 10 mg/kg group showing a marked reduction in tumor size compared with vehicle-treated controls (Fig. 7A, B). Notably, combined SA and MK-6482 treatment produced greater tumor growth inhibition than either monotherapy, which was further confirmed by significantly lower tumor weights at the experimental endpoint (Fig. 7C).

RT-qPCR analysis of excised tumors demonstrated that SA significantly downregulated multiple lipogenic genes, including ACLY, CD36, FABP4, ACC1, FASN, SCD1, and ACSS2 (Fig. 7D-J). Histological analysis revealed no obvious pathological abnormalities in major organs, indicating a favorable safety profile (Fig. 7K). Immunohistochemical staining further showed reduced Ki67 proliferation index together with decreased expression of the lipogenic enzymes FASN and SCD1 in SA-treated tumors. Consistently, Oil Red O staining demonstrated a marked reduction in intratumoral lipid accumulation (Fig. 7L-P). These findings indicate that SA suppresses ccRCC growth in vivo through inhibition of SREBP1-driven lipogenic programs while enhancing the antitumor efficacy of MK-6482.

Immunoblot analysis of xenograft tumors showed that SA dose-dependently inhibited SREBP1 activation, as evidenced by accumulation of precursor SREBP1 and reduced mature SREBP1, accompanied by decreased FASN and ACC1 expression. Combined treatment with MK-6482 further enhanced suppression of VEGFA and SREBP1-driven lipogenic signaling (Supplementary Fig. S7A). To further characterize SA-induced metabolic alterations in vivo, untargeted lipidomic profiling was performed using xenograft tumor tissues. A total of 3,358 metabolites were identified, representing six superclasses, 28 lipid classes, and 96 lipid subclasses, indicating broad metabolic coverage (Supplementary Fig. S7B). Principal component analysis demonstrated clear separation among the control, SA, MK-6482, and combination groups, indicating distinct metabolic profiles following treatment (Supplementary Fig. S7C). Hierarchical clustering further revealed pronounced remodeling of lipid-associated metabolites in SA-treated tumors (Supplementary Fig. S7D).

Comparative lipidomic analyses further demonstrated that MK-6482 or SA treatment significantly remodeled glycerolipid, phospholipids, and sphingolipid species (Supplementary Fig. 7E-F). Notably, SA treatment alone or in combination with MK-6482 led to coordinated reductions in triglycerides and membrane lipid species, accompanied by redistribution of phospholipid and ceramide-related metabolites (Supplementary Fig. 7G-H). These results indicate that SA induces extensive lipid metabolic reprogramming in ccRCC cells, supporting suppression of lipogenic programs.

## Discussion

**Fig. 8:**
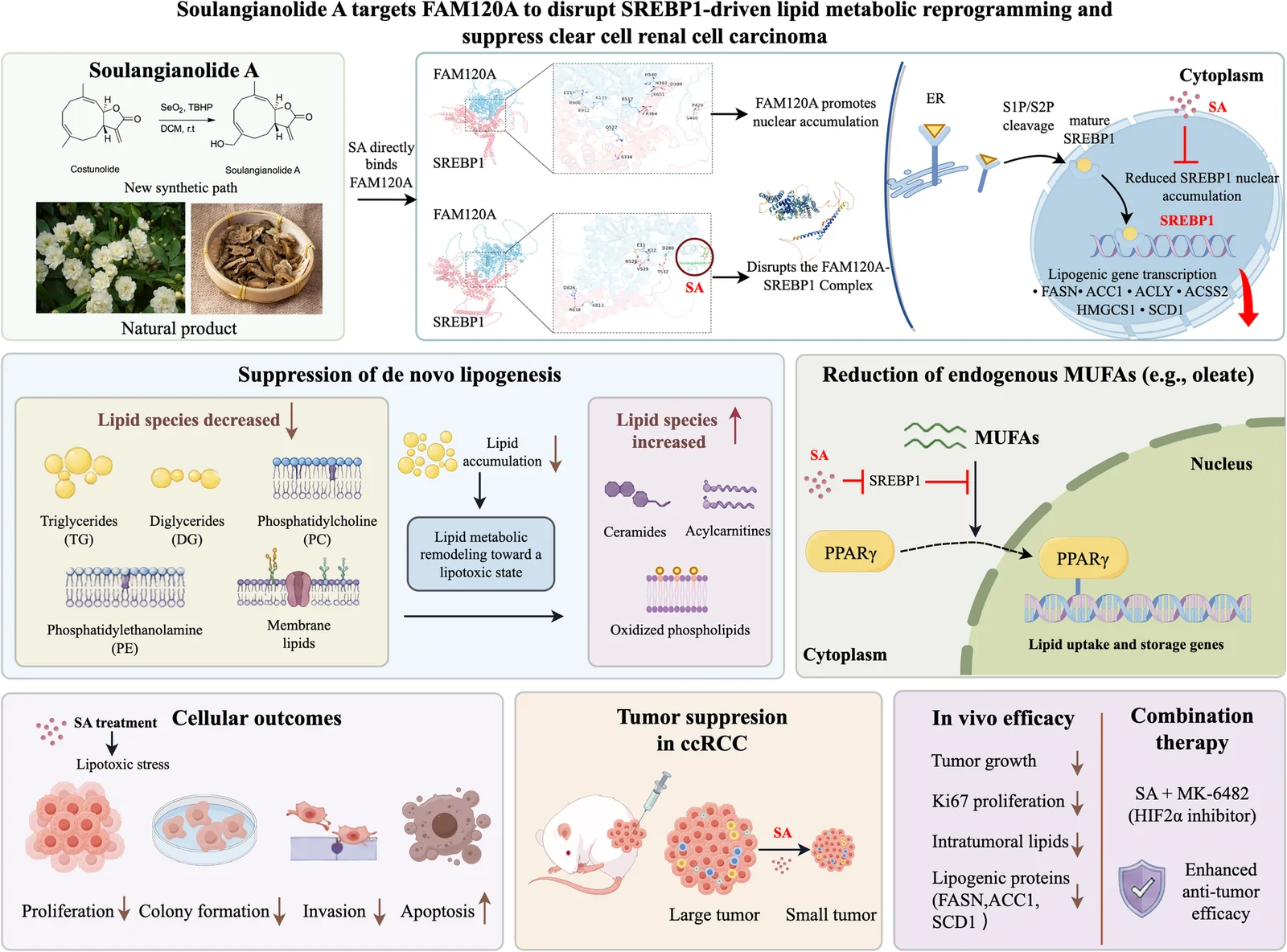
Proposed working model illustrating the mechanism by which Soulangianolide A suppresses ccRCC progression through targeting the FAM120A-SREBP1 axis

In the present study, we identified FAM120A as a previously unrecognized molecular target of Soulangianolide A (SA) and demonstrated that SA suppresses SREBP1-dependent lipid metabolic reprogramming through direct targeting of the FAM120A-SREBP1 axis in clear cell renal cell carcinoma (ccRCC). Mechanistically, SA binds FAM120A through Cys531, disrupts the FAM120A-SREBP1 complex, and thereby impairs SREBP1-dependent lipogenic transcription. As a consequence, inhibition of the FAM120A–SREBP1 axis induces extensive lipid metabolic remodeling, attenuates lipid-dependent PPARγ signaling, suppresses tumor growth, and enhances the antitumor efficacy of HIF-2α inhibition. Collectively, our findings identify the FAM120A-SREBP1 axis as a previously unrecognized metabolic vulnerability in ccRCC and provide a mechanistic rationale for targeting this pathway as a therapeutic strategy (Fig. 8).

Aberrant lipid metabolic reprogramming is a defining hallmark of ccRCC and is essential for supporting tumor growth and progression [31, 32]. SREBP1 is widely recognized as a master regulator of de novo lipogenesis, and its activation has been closely associated with aggressive disease and poor prognosis in ccRCC [33]. Although previous studies have suggested that FAM120A participates in the regulation of SREBP1 signaling and lipid metabolism in certain biological contexts [17], its functional significance in ccRCC has remained largely unexplored, and whether this pathway could be therapeutically targeted has not been investigated. Our findings demonstrate that the FAM120A-SREBP1 axis is a critical driver of lipid metabolic reprogramming in ccRCC. Our findings further show that FAM120A promotes SREBP1-dependent transcriptional activity and lipogenic gene expression, thereby sustaining the metabolic phenotype of ccRCC cells. More importantly, our findings suggest that FAM120A represents a targetable upstream regulator of SREBP1 signaling and that disruption of the FAM120A-SREBP1 interaction effectively suppresses lipid metabolic reprogramming in ccRCC. These findings not only expand our understanding of the role of the FAM120A-SREBP1 axis in ccRCC lipid metabolism but also provide a mechanistic rationale for the development of metabolism-targeted therapeutic strategies.

Direct identification of the molecular target is essential for understanding the mechanism of action of bioactive small molecules. Although biochemical assays such as pull-down, SPR, and CETSA supported the interaction between SA and FAM120A, these approaches alone could not establish residue-level specificity or determine whether direct binding was responsible for the biological activity of SA. Identification of Cys531 as the critical SA-binding residue, together with functional validation using the binding-deficient C531A mutant, establishes a direct mechanistic link between molecular target engagement and inhibition of SREBP1 signaling. Importantly, disruption of this single residue largely abolished the inhibitory effects of SA on the FAM120A-SREBP1 interaction and markedly attenuated its effects on lipogenic signaling and malignant phenotypes, indicating that specific target engagement is a major determinant of the antitumor activity of SA. Collectively, these findings provide direct molecular evidence linking target engagement with downstream biological responses and further support FAM120A as a potential therapeutic target for metabolism-oriented intervention in ccRCC.

FAM120A is a multifunctional scaffold protein that participates in diverse biological processes, including RNA metabolism, oxidative stress responses, and intracellular signaling [18, 34]. In the present study, our findings suggest that FAM120A primarily regulates the transcriptional competence of SREBP1 rather than directly controlling its expression or maturation. FAM120A depletion markedly impaired SREBP1 occupancy at the promoters of multiple lipogenic genes, whereas restoration of constitutively active mature SREBP1 partially rescued lipogenic signaling and malignant phenotypes, indicating that FAM120A functions upstream of SREBP1-dependent transcription. These observations support a model in which FAM120A acts as a scaffold and co-regulatory protein that facilitates efficient SREBP1 transcriptional activity and lipogenic gene expression. Importantly, our data do not support a role for FAM120A in regulating every step of SREBP1 activation; rather, they indicate that FAM120A is primarily required for maintaining efficient nuclear transcriptional activity of SREBP1.

Our expanded lipidomic analyses further demonstrate that SA does not simply reduce intracellular lipid content but instead induces extensive remodeling of the lipidome. Rather than causing a uniform depletion of lipid species, inhibition of the FAM120A-SREBP1 axis resulted in coordinated reductions in storage-associated lipids and membrane phospholipids, accompanied by increased levels of ceramides, acylcarnitines, and oxidized phospholipids. These coordinated changes are indicative of a metabolic transition from an anabolic lipid-storage state toward a lipotoxic state [35–37]. Such remodeling is likely to compromise membrane biogenesis, perturb lipid-dependent signaling, and increase metabolic stress, thereby creating conditions that are unfavorable for sustained tumor growth [38]. Importantly, these lipidomic alterations are consistent with the established role of SREBP1 as a master regulator of de novo lipogenesis and provide metabolic evidence that complements our molecular and genetic findings. Collectively, these observations suggest that therapeutic targeting of the FAM120A–SREBP1 axis not only suppresses lipid biosynthesis but also reshapes the lipid metabolic landscape in a manner that limits the metabolic adaptability of ccRCC cells. This extensive lipidomic remodeling may also have broader consequences for lipid-dependent signaling pathways. Indeed, multiple lipid species generated downstream of SREBP1 serve as endogenous activators of lipid-responsive transcription factors, providing a mechanistic basis for the secondary suppression of PPARγ signaling observed following SA treatment.

PPARγ is a lipid-sensing nuclear receptor whose transcriptional activity depends on endogenous fatty acid ligands and plays an important role in lipid uptake, storage, and metabolic homeostasis [23, 39]. Although the role of PPARγ in ccRCC remains controversial [40, 41], with inconsistent associations reported between its expression and clinical outcome, these discrepancies may partly reflect the fact that PPARγ activity is largely determined by ligand availability and nuclear transcriptional activity rather than transcript abundance alone [39]. Our findings support a hierarchical regulatory model in which inhibition of the FAM120A-SREBP1 axis suppresses endogenous fatty acid synthesis, thereby limiting ligand availability for PPARγ activation. Consistent with this model, SREBP1 depletion markedly reduced nuclear localization of PPARγ, whereas exogenous oleic acid restored nuclear PPARγ and downstream target gene expression without reactivating SREBP1. These findings indicate that attenuation of PPARγ signaling represents a downstream metabolic consequence of impaired SREBP1-dependent lipogenesis rather than a direct effect of SA on PPARγ.

Our findings highlight the therapeutic potential of targeting the FAM120A–SREBP1 axis in ccRCC. Although immune checkpoint inhibitors and HIF-2α inhibitors have substantially improved the treatment of advanced ccRCC, therapeutic resistance remains a major clinical challenge, highlighting the need for additional metabolism-targeted therapeutic strategies [28, 42, 43]. In this context, disruption of the FAM120A-SREBP1 axis represents a promising approach for suppressing lipid metabolic reprogramming in ccRCC. Importantly, the enhanced antitumor efficacy observed following combined treatment with SA and the HIF-2α inhibitor MK-6482 further supports the concept that simultaneous targeting of hypoxia signaling and metabolic dependency may provide greater therapeutic benefit than either strategy alone. This observation is consistent with previous evidence indicating functional crosstalk between hypoxia signaling and lipid metabolism in ccRCC. In addition, because SA is present only at extremely low abundance in its natural source, we established a practical chemical synthetic route to ensure sufficient compound availability for mechanistic investigation and future drug development. Although additional pharmacokinetic, toxicological, and preclinical studies will be required before clinical translation, our findings provide a strong rationale for further exploration of FAM120A-targeting strategies as a potential therapeutic approach for ccRCC.

Despite the strengths of the present study, several limitations should also be acknowledged. Although our data demonstrate that FAM120A promotes SREBP1-dependent chromatin occupancy and transcriptional activation, the precise molecular mechanism by which FAM120A facilitates SREBP1 recruitment to chromatin remains to be fully elucidated. Further structural and biochemical studies will help define the molecular basis of this regulatory process. In addition, although the antitumor activity of SA was validated in xenograft models, further evaluation in patient-derived models and larger clinical cohorts will be important to further validate the therapeutic potential of targeting the FAM120A-SREBP1 axis. Finally, comprehensive pharmacokinetic, pharmacodynamic, and toxicological studies will be required before clinical translation of SA. Despite these limitations, our findings provide a rationale for targeting the FAM120A-SREBP1 axis represents a promising strategy for disrupting lipid metabolic reprogramming in ccRCC and lay the foundation for the future development of metabolism-targeted therapies.

## Conclusion

SA directly targets FAM120A to disrupt SREBP1-dependent lipogenic signaling, thereby limiting lipid-dependent activation of PPARγ and inducing tumor suppression through metabolic stress. These findings identify the FAM120A-SREBP1-PPARγ axis as a critical metabolic vulnerability in ccRCC, establish FAM120A as a druggable regulator of lipid metabolic reprogramming, and highlight SA as a promising metabolism-targeted therapeutic strategy for ccRCC.

## Supplementary Information

Supplementary Material 1.

Supplementary Material 2.

## Data Availability

No datasets were generated or analysed during the current study.

## References

[1] Hsieh J J, Purdue M P, Signoretti S, et al. Renal cell carcinoma [J]. Nat Rev Dis Primers. 2017;3:17009. 10.1038/nrdp.2017.9 PMC5936048 28276433

[2] Siegel R L, Miller K D, Fuchs H E, et al. Cancer Statistics, 2021 [J]. CA Cancer J Clin. 2021;71(1):7–33. 10.3322/caac.21654 33433946

[3] Leibovich B C, Lohse C M, Cheville JC, et al. Predicting Oncologic Outcomes in Renal Cell Carcinoma After Surgery [J]. Eur Urol. 2018;73(5):772–80. 10.1016/j.eururo.2018.01.005 29398265

[4] Valera V A, Merino MJ. Misdiagnosis of clear cell renal cell carcinoma [J]. Nat Rev Urol. 2011;8(6):321–33. 10.1038/nrurol.2011.64 21587224

[5] Riscal R, Bull C J, Mesaros C, et al. Cholesterol Auxotrophy as a Targetable Vulnerability in Clear Cell Renal Cell Carcinoma [J]. Cancer Discov. 2021;11(12):3106–25. 10.1158/2159-8290.CD-21-0211 PMC8741905 34244212

[6] Wettersten H I, Aboud O A, Lara P N JR, et al. Metabolic reprogramming in clear cell renal cell carcinoma [J]. Nat Rev Nephrol. 2017;13(7):410–9. 10.1038/nrneph.2017.59 28480903

[7] Zhang Y, Zhang S, Sun H, et al. The pathogenesis and therapeutic implications of metabolic reprogramming in renal cell carcinoma [J]. Cell Death Discov. 2025;11(1):186. 10.1038/s41420-025-02479-9 PMC12009291 40253354

[8] Hu J, Wang S G, Hou Y, et al. Multi-omic profiling of clear cell renal cell carcinoma identifies metabolic reprogramming associated with disease progression [J]. Nat Genet. 2024;56(3):442–57. 10.1038/s41588-024-01662-5 PMC10937392 38361033

[9] Marandino L, Campi R, Amparore D, et al. Neoadjuvant and Adjuvant Immune-based Approach for Renal Cell Carcinoma: Pros, Cons, and Future Directions [J]. Eur Urol Oncol. 2025;8(2):494–509. 10.1016/j.euo.2024.09.002 39327187

[10] Bex A, Ghanem Y A, Albiges L, et al. European Association of Urology Guidelines on Renal Cell Carcinoma: The 2025 Update [J]. Eur Urol. 2025;87(6):683–96. 10.1016/j.eururo.2025.02.020 40118739

[11] He Y, Qi S, Chen L. The roles and mechanisms of SREBP1 in cancer development and drug response [J]. Genes Dis. 2024;11(4):100987. 10.1016/j.gendis.2023.04.022 PMC10978545 38560498

[12] Lao M, Zhang X, Li Z, et al. Lipid metabolism reprograming by SREBP1-PCSK9 targeting sensitizes pancreatic cancer to immunochemotherapy [J]. Cancer Commun (Lond). 2025;45(8):1010–37. 10.1002/cac2.70038 PMC12766276 40439109

[13] Chen M, Li H, Zheng S, et al. Nobiletin targets SREBP1/ACLY to induce autophagy-dependent cell death of gastric cancer cells through PI3K/Akt/mTOR signaling pathway [J]. Phytomedicine. 2024;128:155360. 10.1016/j.phymed.2024.155360 38547624

[14] Osborne T F, Espenshade P J. Evolutionary conservation and adaptation in the mechanism that regulates SREBP action: what a long, strange tRIP it’s been [J]. Genes Dev. 2009;23(22):2578–91. 10.1101/gad.1854309 PMC2779761 19933148

[15] Horton J D, Goldstein J L, Brown M S. SREBPs: activators of the complete program of cholesterol and fatty acid synthesis in the liver [J]. J Clin Invest. 2002;109(9):1125–31. 10.1172/JCI15593 PMC150968 11994399

[16] Guo D, Bell E H, Mischel P, et al. Targeting SREBP-1-driven lipid metabolism to treat cancer [J]. Curr Pharm Des. 2014;20(15):2619–26. 10.2174/13816128113199990486 PMC4148912 23859617

[17] Cho S, Chun Y, He L, et al. FAM120A couples SREBP-dependent transcription and splicing of lipogenesis enzymes downstream of mTORC1 [J]. Mol Cell. 2023;83(16):3010–e268. 10.1016/j.molcel.2023.07.017 PMC10494788 37595559

[18] Niu L, Li Y, Huang G, et al. FAM120A deficiency improves resistance to cisplatin in gastric cancer by promoting ferroptosis [J]. Commun Biol. 2024;7(1):399. 10.1038/s42003-024-06097-6 PMC10987584 38565940

[19] Shi W L, Wang J, Ma Y, et al. [Advances in research methods for biosynthetic pathway analysis of active ingredients in traditional Chinese medicine] [J]. Zhongguo Zhong Yao Za Zhi. 2023;48(9):2273–83. 10.19540/j.cnki.cjcmm.20230324.101 37282856

[20] Naeem A, Hu P, Yang M, et al. Natural Products as Anticancer Agents: Current Status and Future Perspectives [J]. Molecules. 2022;27(23):8367. 10.3390/molecules27238367 PMC9737905 36500466

[21] Chen J, Zhao Z, Lin L, et al. Aucklandia lappa Decne: a review of its botany, cultivation, ethnopharmacology, phytochemistry, pharmacology, and practical applications [J]. Front Pharmacol. 2025;16:1659831. 10.3389/fphar.2025.1659831 PMC12554694 41158126

[22] Luo W, Song Z, Xu G, et al. LicochalconeB inhibits cGAS-STING signaling pathway and prevents autoimmunity diseases [J]. Int Immunopharmacol. 2024;128:111550. 10.1016/j.intimp.2024.111550 38232536

[23] Hernandez-Quiles M, Broekema M F, Kalkhoven E. PPARgamma in Metabolism, Immunity, and Cancer: Unified and Diverse Mechanisms of Action [J]. Front Endocrinol (Lausanne). 2021;12:624112. 10.3389/fendo.2021.624112 PMC7953066 33716977

[24] Chen H, Tan H, Wan J, et al. PPAR-γ signaling in nonalcoholic fatty liver disease: Pathogenesis and therapeutic targets [J]. Pharmacol Ther. 2023;245:108391. 10.1016/j.pharmthera.2023.108391 36963510

[25] Kounatidis D, Vallianou N G, Rebelos E, et al. The Many Facets of PPAR-γ Agonism in Obesity and Associated Comorbidities: Benefits, Risks, Challenges, and Future Directions [J]. Curr Obes Rep. 2025;14(1):19. 10.1007/s13679-025-00612-4 39934485

[26] Jonasch E, Donskov F, Iliopoulos O, et al. Belzutifan for Renal Cell Carcinoma in von Hippel-Lindau Disease [J]. N Engl J Med. 2021;385(22):2036–46. 10.1056/NEJMoa2103425 PMC9275515 34818478

[27] Choi W W, Boland J L, Kalola A, et al. Belzutifan (MK-6482): Biology and Clinical Development in Solid Tumors [J]. Curr Oncol Rep. 2023;25(2):123–9. 10.1007/s11912-022-01354-5 36630072

[28] Choueiri T K, Powles T, Peltola K, et al. Belzutifan versus Everolimus for Advanced Renal-Cell Carcinoma [J]. N Engl J Med. 2024;391(8):710–21. 10.1056/NEJMoa2313906 39167807

[29] Liao C, Hu L, Zhang Q. Von Hippel-Lindau protein signalling in clear cell renal cell carcinoma [J]. Nat Rev Urol. 2024;21(11):662–75. 10.1038/s41585-024-00876-w 38698165

[30] Hua X, Ge S, Zhang L, et al. MED15 is upregulated by HIF-2α and promotes proliferation and metastasis in clear cell renal cell carcinoma via activation of SREBP-dependent fatty acid synthesis [J]. Cell Death Discov. 2024;10(1):188. 10.1038/s41420-024-01944-1 PMC11035615 38649345

[31] Wettersten H I, Aboud O A, Lara P N, et al. Metabolic reprogramming in clear cell renal cell carcinoma [J]. Nat Rev Nephrol. 2017;13(7):410–9. 10.1038/nrneph.2017.59 28480903

[32] Hakimi A A, Reznik E, Lee C H, et al. An Integrated Metabolic Atlas of Clear Cell Renal Cell Carcinoma [J]. Cancer Cell. 2016;29(1):104–16. 10.1016/j.ccell.2015.12.004 PMC4809063 26766592

[33] Shen D, Gao Y, Huang Q, et al. E2F1 promotes proliferation and metastasis of clear cell renal cell carcinoma via activation of SREBP1-dependent fatty acid biosynthesis [J]. Cancer Lett. 2021;514:48–62. 10.1016/j.canlet.2021.05.012 34019961

[34] Bartolomé R A, García-Palmero I, Torres S, et al. IL13 Receptor α2 Signaling Requires a Scaffold Protein, FAM120A, to Activate the FAK and PI3K Pathways in Colon Cancer Metastasis [J]. Cancer Res. 2015;75(12):2434–44. 10.1158/0008-5472.CAN-14-3650 25896327

[35] Chaurasia B, Summers S A. Ceramides in Metabolism: Key Lipotoxic Players [J]. Annu Rev Physiol. 2021;83:303–30. 10.1146/annurev-physiol-031620-093815 PMC7905841 33158378

[36] Wajapeyee N, Beamon T C, Gupta R. Roles and therapeutic targeting of ceramide metabolism in cancer [J]. Mol Metab. 2024;83:101936. 10.1016/j.molmet.2024.101936 PMC11031839 38599378

[37] Vogel F C E, Chaves-Filho A B, Schulze A. Lipids as mediators of cancer progression and metastasis [J]. Nat Cancer. 2024;5(1):16–29. 10.1038/s43018-023-00702-z 38273023

[38] Vasseur S, Guillaumond F. Lipids in cancer: a global view of the contribution of lipid pathways to metastatic formation and treatment resistance [J]. Oncogenesis. 2022;11(1):46. 10.1038/s41389-022-00420-8 PMC9363460 35945203

[39] Montaigne D, Butruille L, Staels B. PPAR control of metabolism and cardiovascular functions [J]. Nat Rev Cardiol. 2021;18(12):809–23. 10.1038/s41569-021-00569-6 34127848

[40] Sanchez D J, Steger D J, Skuli N, et al. PPARγ is dispensable for clear cell renal cell carcinoma progression [J]. Mol Metab. 2018;14:139–49. 10.1016/j.molmet.2018.05.013 PMC6034040 29866440

[41] Petitprez F, Ayadi M, De Reyniès A, et al. Review of Prognostic Expression Markers for Clear Cell Renal Cell Carcinoma [J]. Front Oncol. 2021;11:643065. 10.3389/fonc.2021.643065 PMC8113694 33996558

[42] Choueiri T K, Powles T, Burotto M, et al. Nivolumab plus Cabozantinib versus Sunitinib for Advanced Renal-Cell Carcinoma [J]. N Engl J Med. 2021;384(9):829–41. 10.1056/NEJMoa2026982 PMC8436591 33657295

[43] Gkolfinopoulos S, Psyrri A, Bamias A. Clear-cell renal cell carcinoma - A comprehensive review of agents used in the contemporary management of advanced/metastatic disease [J]. Oncol Rev. 2021;15(1):530. 10.4081/oncol.2021.530 PMC7967495 33747368

