# Supplementary material for: Soulangianolide A targets FAM120A to disrupt SREBP1-driven lipid metabolic reprogramming and suppress clear cell renal cell carcinoma
Source: J Exp Clin Cancer Res. 2026 Jul 17;45:215. doi: 10.1186/s13046-026-03788-2 (PMC13660240; doi:10.1186/s13046-026-03788-2)
Supplement: Supplementary file 1 [file 13046_2026_3788_MOESM1_ESM.pdf]

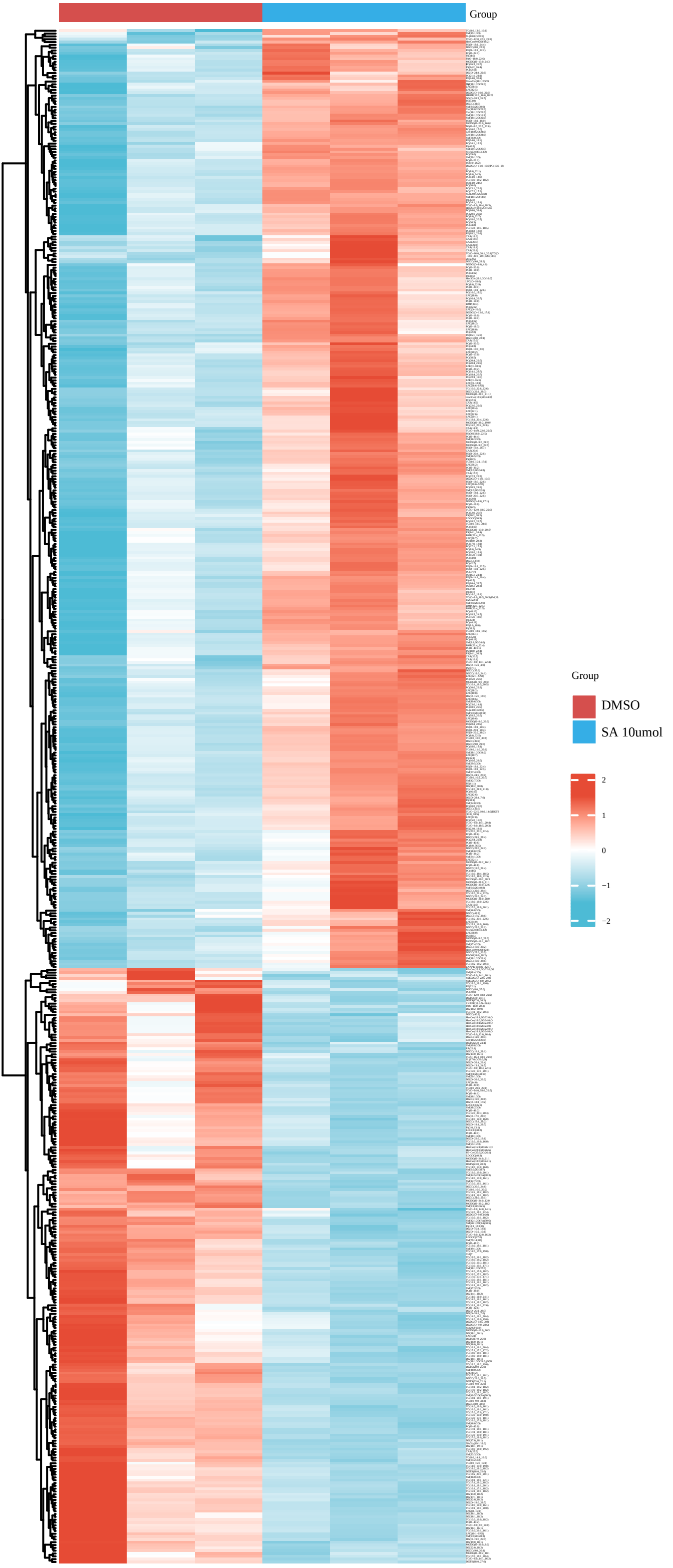
Group
Group
DMSO
SA 10umol
2
1
0
−1
−2
