# Supplementary material for: Soulangianolide A targets FAM120A to disrupt SREBP1-driven lipid metabolic reprogramming and suppress clear cell renal cell carcinoma
Source: J Exp Clin Cancer Res. 2026 Jul 17;45:215. doi: 10.1186/s13046-026-03788-2 (PMC13660240; doi:10.1186/s13046-026-03788-2)
Supplement: Supplementary file 2 [file 13046_2026_3788_MOESM2_ESM.docx]

**Supplementary Table S1.**

| SREBF1(Human) | Forward | [ACAGTGACTTCCCTGGCCTAT](https://journals.plos.org/plosone/article?id=10.1371/journal.pone.0259482) |
| --- | --- | --- |
|  | Reverse | GCATGGACGGGTACATCTTCAA |
| FASN(Human) | Forward | [TTCTACGGCTCCACGCTCTTCC](https://www.origene.com/catalog/gene-expression/qpcr-primer-pairs/hp207467-fatty-acid-synthase-fasn-human-qpcr-primer-pair-nm-004104) |
|  | Reverse | GAAGAGTCTTCGTCAGCCAGGA |
| SCD1(Human) | Forward | CCTGGTTTCACTTGGAGCTGTG |
|  | Reverse | TGTGGTGAAGTTGATGTGCCAGC |
| ACLY(Human) | Forward | TCGGCCAAGGCAATTTCAGAG |
|  | Reverse | CGAGCATACTTGAACCGATTCT |
| ACSS2(Human) | Forward | AAAGGAGCAACTACCAACATCTG |
|  | Reverse | GCTGAACTGACACACTTGGAC |
| HMGCS1(Human) | Forward | GATGTGGGAATTGTTGCCCTT |
|  | Reverse | ATTGTCTCTGTTCCAACTTCCAG |
| FABP4(Human) | Forward | ACGAGAGGATGATAAACTGGTGG |
|  | Reverse | GCGAACTTCAGTCCAGGTCAAC |
| MVD(Human) | Forward | CTCCCTGAGCGTCACTCTG |
|  | Reverse | GGTCCTCGGTGAAGTCCTTG |
| FDFT1(Human) | Forward | GCAACGCAGTGTGCATATTTT |
|  | Reverse | CGCCAGTCTGGTTGGTAAAGG |
| ACC1(Human) | Forward | TTCACTCCACCTTGTCAGCGGA |
|  | Reverse | GTCAGAGAAGCAGCCCATCACT |
| CD36(Human) | Forward | CAGGTCAACCTATTGGTCAAGCC |
|  | Reverse | GCCTTCTCATCACCAATGGTCC |
| LPL(Human) | Forward | CTGCTGGCATTGCAGGAAGTCT |
|  | Reverse | CATCAGGAGAAAGACGACTCGG |
| SCD1(Human) | Forward | CCGGGAGAATATCCTGGTTT |
|  | Reverse | GCGGTACTCAACTGGCAGAGT |
| GAPDH(Human) | Forward | GGAGCGAGATCCCTCCAAAAT |
|  | Reverse | GGCTGTTGTCATACTTCTCATGG |
| ACLY(mouse) | Forward | AGGACCTGTCTGCTGTTGTT |
|  | Reverse | TCTTGGTGATGGTGGTCTTG |
| CD36(mouse) | Forward | ATGGGCTGTGATCGGAACTG |
|  | Reverse | GTCTTCCCAATAAGCATGTCTCC |
| FABP4(mouse) | Forward | AAGGTGAAGAGCATCATAACCCT |
|  | Reverse | TCACGCCTTTCATAACACATTCC |
| ACC1 (mouse) | Forward | TGGCAGCTTTGGAGGTGTAT |
|  | Reverse | TGGAGTGTTCTGTCCAGTGG |
| FASN(mouse) | Forward | GGAGGTGGTGATAGCCGGTAT |
|  | Reverse | TGGGTAATCCATAGAGCCCAG |
| SCD1(mouse) | Forward | TTCTTGCGATACACTCTGGTGC |
|  | Reverse | CGGGATTGAATGTTCTTGTCGT |
| ACSS2(mouse) | Forward | AGGCTGCTACTGGAAGATGG |
|  | Reverse | CAGGATGGTGATGTTGGTGA |
| GAPDH(mouse) | Forward | AGGTCGGTGTGAACGGATTTG |
|  | Reverse | TGTAGACCATGTAGTTGAGGTCA |

Supplementary Table S2.

| **REAGENT or RESOURCE** | **SOURCE** | **IDENTIFIER** |
| --- | --- | --- |
| SREBP1 | Proteintech | 14088-1-AP |
| SREBP1 | Santa Cruz Biotechnology | sc-8984 |
| FAM120A | GeneTex | GTX120824 |
| FAM120A | Thermo Fisher Scientific | A303-889A |
| FASN | Proteintech | 10624-2-AP |
| ACC1 | Proteintech | 21923-1-AP |
| ACLY | Proteintech | 15421-1-AP |
| ACSS2 | Proteintech | 15966-1-AP |
| SCD1 | Proteintech | 23393-1-AP |
| CD36 | Proteintech | 18836-1-AP |
| PPARγ | Proteintech | 16643-1-AP |
| Ki67 | Proteintech | 27309-1-AP |
| GAPDH | Proteintech | 10494-1-AP |
| HSP90 | Proteintech | 13171-1-AP |
| IgG | Cell Signaling Technology | 2729 |
| Lamin B1 | Proteintech | 12987-1-AP |
| Tubulin | Proteintech | 14555-1-AP |
| AntiFLAG M2  Magnetic Beads | Sigma | M8823 |

**Supplementary Figure S1. Quantification of histological analyses in SREBP1-knockdown xenografts.**

(A-D) Quantification of immunohistochemical staining intensity (AOD) for SREBP1, FASN, ACC1, and SCD1 in xenograft tumors. (E) Quantification of Oil Red O-positive area. (F) Quantification of Ki67-positive cells.Data are presented as mean ± SD (n = 5 mice per group). Statistical significance was determined using an unpaired two-tailed Student's *t*-test. ***P < 0.001.

**Supplementary Fig. S2. Screening, synthesis, and structural validation of SA**
**(A)** Representative immunofluorescence images of SREBP1 staining and TUNEL assay in 786-O cells treated with DMSO or SA (10 μmol/L). DAPI was used for nuclear staining. **(B)** Synthetic route of SA from costunolide and structural validation by ^1H NMR and ^13C NMR spectroscopy (600 MHz).

**Supplementary Fig. S3. FAM120A preferentially interacts with SREBP1 and promotes SREBP1-dependent lipogenic transcription.**

(A) Co-immunoprecipitation analysis showing the interaction of Flag-tagged FAM120A with m-SREBP1, mature SREBP2 (m-SREBP2), ChREBP, PPARγ, and p300 in 786-O cells. (B) Co-immunoprecipitation analysis showing that SA selectively disrupts the interaction between FAM120A and SREBP1 but has little effect on its interaction with SRSF1, RNA Polymerase II, AGO2, and U1-70K. (C) Co-immunoprecipitation analysis showing that SA preferentially disrupts the interaction between FAM120A and SREBP1 compared with other lipid metabolism-related transcription factors, including SREBP2, ChREBP, LXRα, and LXRβ. (D) Primer sequences used for ChIP-qPCR analysis of the ACC1, SCD1, and ACLY promoter regions. (E) ChIP-qPCR analysis showing reduced SREBP1 occupancy at the promoters of FASN, ACC1, and SCD1 following FAM120A knockdown. (F) Representative ChIP-PCR analysis confirming decreased SREBP1 binding to the promoters of FASN, ACC1, and SCD1 after FAM120A depletion. (G) Immunoblot analysis showing that ectopic expression of mature SREBP1 partially restores the expression of downstream lipogenic proteins suppressed by FAM120A knockdown in 786-O and Caki-1 cells. Data are representative of three independent biological experiments unless otherwise indicated.

**Supplementary Fig. S4. Cys531 of FAM120A is required for SA-mediated suppression of invasion and proliferation in ccRCC cells.**

**(A)** Representative Transwell invasion assays of 786-O and Caki-1 cells treated with DMSO, SA (10 μM), WT-FAM120A, WT-FAM120A + SA, C531A-FAM120A, or C531A-FAM120A + SA. **(B-C)** Quantification of invaded cells in 786-O (**B**) and Caki-1 (**C**) cells under the indicated conditions. **(D)** (D) Representative EdU incorporation assays of 786-O and Caki-1 cells under the indicated treatments (scale bar, 50 μm). **(E-F)** Quantification of EdU-positive cells in 786-O (**E**) and Caki-1 (**F**) cells.**Data are presented as mean ± SD from three independent biological experiments. Statistical significance was determined by one-way ANOVA.** ***P < 0.001**.**

**Supplementary Fig. S5. Mature SREBP1 partially rescues the metabolic and antitumor effects of SA treatment or FAM120A depletion.**

(A) Cell viability analysis of 786-O and Caki-1 cells treated with DMSO, SA (10 μM), OE-m-SREBP1, or OE-m-SREBP1 plus SA.(B) Representative Oil Red O staining images showing intracellular lipid accumulation under the indicated conditions. (C) Immunoblot analysis of mature SREBP1 (m-SREBP1) and downstream lipogenic proteins (FASN, ACC1, ACLY, and ACSS2) in 786-O and Caki-1 cells under the indicated treatments. (D-E) Representative colony formation assays (D) and quantification of colony numbers (E) in cells treated with DMSO, SA (10 μM), OE-m-SREBP1, or OE-m-SREBP1 plus SA. (F) Representative Oil Red O staining images showing intracellular lipid accumulation in control, sh-FAM120A, sh-FAM120A + OE-m-SREBP1, and sh-FAM120A + OE-m-SREBP1 + SA-treated cells. (G) Quantification of Oil Red O staining. (H-I) Quantification of intracellular triglyceride (H) and total cholesterol (I) levels under the indicated conditions. (J) Cell viability analysis of 786-O and Caki-1 cells following FAM120A depletion with or without OE-m-SREBP1 expression and SA treatment. Data are presented as mean ± SD from three independent biological experiments unless otherwise indicated. Statistical significance was determined by one-way ANOVA. NS, not significant; ***P < 0.001.

**Supplementary Fig. S6. SA induces global transcriptional remodeling associated with metabolic suppression and stress-response activation in ccRCC cells.**

**(A)** KEGG pathway enrichment analysis of differentially expressed genes (DEGs) identified following SA treatment, showing significant enrichment of metabolic and cancer-related pathways. **(B)** KEGG functional classification of DEGs in SA-treated cells. **(C)** Hierarchical clustering heatmap of representative DEGs showing distinct transcriptional profiles between DMSO- and SA-treated ccRCC cells. **(D)** Volcano plot showing significantly upregulated and downregulated genes following SA treatment. **Data were obtained from RNA sequencing analysis of ccRCC cells treated with DMSO or SA (10 μM). Differentially expressed genes were defined as those with |log₂ fold change| > 1 and P < 0.05.**

**Supplementary Fig. S7. SA induces extensive lipid metabolic remodeling and suppresses SREBP1-dependent lipogenic signaling in vivo.**

(A) Immunoblot analysis of VEGFA, p-SREBP1, m-SREBP1, and the downstream lipogenic proteins FASN and ACC1 in xenograft tumors from the indicated treatment groups. GAPDH served as the loading control. (B) Overview of the lipidomic dataset obtained from xenograft tumor tissues, showing the distribution of detected metabolites across superclasses, classes, and subclasses. (C) Principal component analysis (PCA) of lipidomic profiles from control, SA-treated, MK-6482-treated, and combination-treated xenograft tumors. (D) Hierarchical clustering heatmap showing global lipidomic differences among treatment groups. (E-H) Heatmaps showing representative differential lipid species identified in pairwise comparisons between control and SA (10 mg/kg) (E), control and MK-6482 + SA-treated tumors (F), MK-6482 and SA (10 mg/kg)-treated tumors (G), and SA (10 mg/kg)-treated versus MK-6482 + SA-treated tumors (H).
